# Profiling the long noncoding RNA interaction network in the regulatory elements of target genes by chromatin in situ reverse transcription sequencing

**DOI:** 10.1101/gr.244996.118

**Published:** 2019-09

**Authors:** Shilin Zhang, Yichen Wang, Lin Jia, Xue Wen, Zhonghua Du, Cong Wang, Yajing Hao, Dehai Yu, Lei Zhou, Naifei Chen, Jingcheng Chen, Huiling Chen, Hui Zhang, Ilkay Celik, Günhan Gülsoy, Jianjun Luo, Baoming Qin, Xueling Cui, Zhonghui Liu, Songling Zhang, Miguel A. Esteban, Ferhat Ay, Wei Xu, Runsheng Chen, Wei Li, Andrew R. Hoffman, Ji-Fan Hu, Jiuwei Cui

**Affiliations:** 1 Key Laboratory of Organ Regeneration and Transplantation of Ministry of Education, Cancer Center, The First Hospital of Jilin University, Changchun, Jilin 130061, P.R. China;; 2 Stanford University Medical School, VA Palo Alto Health Care System, Palo Alto, California 94304, USA;; 3 CAS Key Laboratory of RNA Biology, Institute of Biophysics, Chinese Academy of Sciences, Beijing 100101, P.R. China;; 4 Department of Endocrinology, Xiangya Hospital, Central South University, Changsha, Hunan 410008, P.R. China;; 5 Guangzhou Institutes of Biomedicine and Health, Chinese Academy of Sciences, Guangzhou, Guangdong 510530, P.R. China;; 6 Google Incorporated, Mountain View, California 94043, USA;; 7 Department of Immunology, College of Basic Medical Sciences, Jilin University, Changchun, Jilin 130021, P.R. China;; 8 La Jolla Institute for Allergy and Immunology, La Jolla, California 92037, USA

## Abstract

Long noncoding RNAs (lncRNAs) can regulate the activity of target genes by participating in the organization of chromatin architecture. We have devised a “chromatin-RNA in situ reverse transcription sequencing” (CRIST-seq) approach to profile the lncRNA interaction network in gene regulatory elements by combining the simplicity of RNA biotin labeling with the specificity of the CRISPR/Cas9 system. Using gene-specific gRNAs, we describe a pluripotency-specific lncRNA interacting network in the promoters of *Sox2* and *Pou5f1*, two critical stem cell factors that are required for the maintenance of pluripotency. The promoter-interacting lncRNAs were specifically activated during reprogramming into pluripotency. Knockdown of these lncRNAs caused the stem cells to exit from pluripotency. In contrast, overexpression of the pluripotency-associated lncRNA activated the promoters of core stem cell factor genes and enhanced fibroblast reprogramming into pluripotency. These CRIST-seq data suggest that the *Sox2* and *Pou5f1* promoters are organized within a unique lncRNA interaction network that determines the fate of pluripotency during reprogramming. This CRIST approach may be broadly used to map lncRNA interaction networks at target loci across the genome.

Nuclear architecture is organized as a highly dynamic structure in a cell-type–specific manner and is tightly coupled with gene transcription (Cavalli and Misteli 2013; Sawyer and Dundr 2017; Wang et al. 2017; Watson and Tsai 2017). Chromatin domains can be subdivided into transcriptionally active and inactive territories based on the status of gene expression (Zullo et al. 2012; Bonev and Cavalli 2016; Tyagi et al. 2016). During cell transitions in development, such as pluripotent reprogramming, chromatin structure may undergo global remodeling in parallel with alterations in gene expression and function (Bhattacharya et al. 2009; Thorpe and Lee 2017). Consequently, genes may be epigenetically turned on or switched off through modifications in the architecture of chromatin DNA, e.g., from transcriptionally active (open) to transcriptionally silent (closed) epigenetic states, or vice versa.

Long noncoding RNAs (lncRNAs) play a critical role in organizing the three-dimensional genome architecture and regulating gene activity in *cis* or in *trans* through multiple mechanisms (Chen and Carmichael 2010; Batista and Chang 2013; Werner and Ruthenburg 2015; Chédin 2016; Huang et al. 2016; Qin et al. 2016; Sridhar et al. 2017; Lan et al. 2018). For example, the genome of pluripotent stem cells is organized in the form of higher-order chromatin architecture, with a variety of intra- and interchromosomal interactions, depending on the status of pluripotency (Denholtz et al. 2013; Sexton and Cavalli 2013; Ji et al. 2016). The nuclear architecture around the promoter region of stem cell core factor genes directly determines the fate of stem cell pluripotency (Kagey et al. 2010; Apostolou et al. 2013; Wei et al. 2013; Zhang et al. 2013). In most cases, the chromatin structure is composed of chromatin DNA loops, lncRNAs, and protein factors that control the transcriptional program to establish the stemness state (Phillips-Cremins et al. 2013). Functionally, this lncRNA chromatin structure may bring distant enhancer elements into proximity of the core promoter (Zhang et al. 2013; Li et al. 2014; Sun et al. 2014; Wang et al. 2015).

To profile the lncRNA regulatory network at a specific gene locus, we developed a “chromatin RNA in situ reverse transcription-associated sequencing” (CRIST-seq) assay. As a proof of concept, we utilized CRIST-seq to profile lncRNAs that interact with the promoter complexes of *Sox2* and *Pou5f1*, two critical core stem cell factors required for pluripotent reprogramming. We have discovered a pluripotency-specific lncRNA interaction network in the *Sox2* and *Pou5f1* promoters, in which the promoter-interacting lncRNAs were closely associated with pluripotency during reprogramming. To determine the broad application of this CRIST assay, we also mapped noncoding RNAs in tumor-associated genes, including the proto-oncogene *FLI1* and the fetal mitogen insulin-like growth factor II (*IGF2*). Because of the flexibility of gene-specific gRNAs, this CRIST-seq approach may be used broadly to map lncRNA interaction networks at target loci across the genome.

## Results

### Mapping chromatin lncRNAs in gene regulatory elements by CRIST-seq

**Figure 1.**
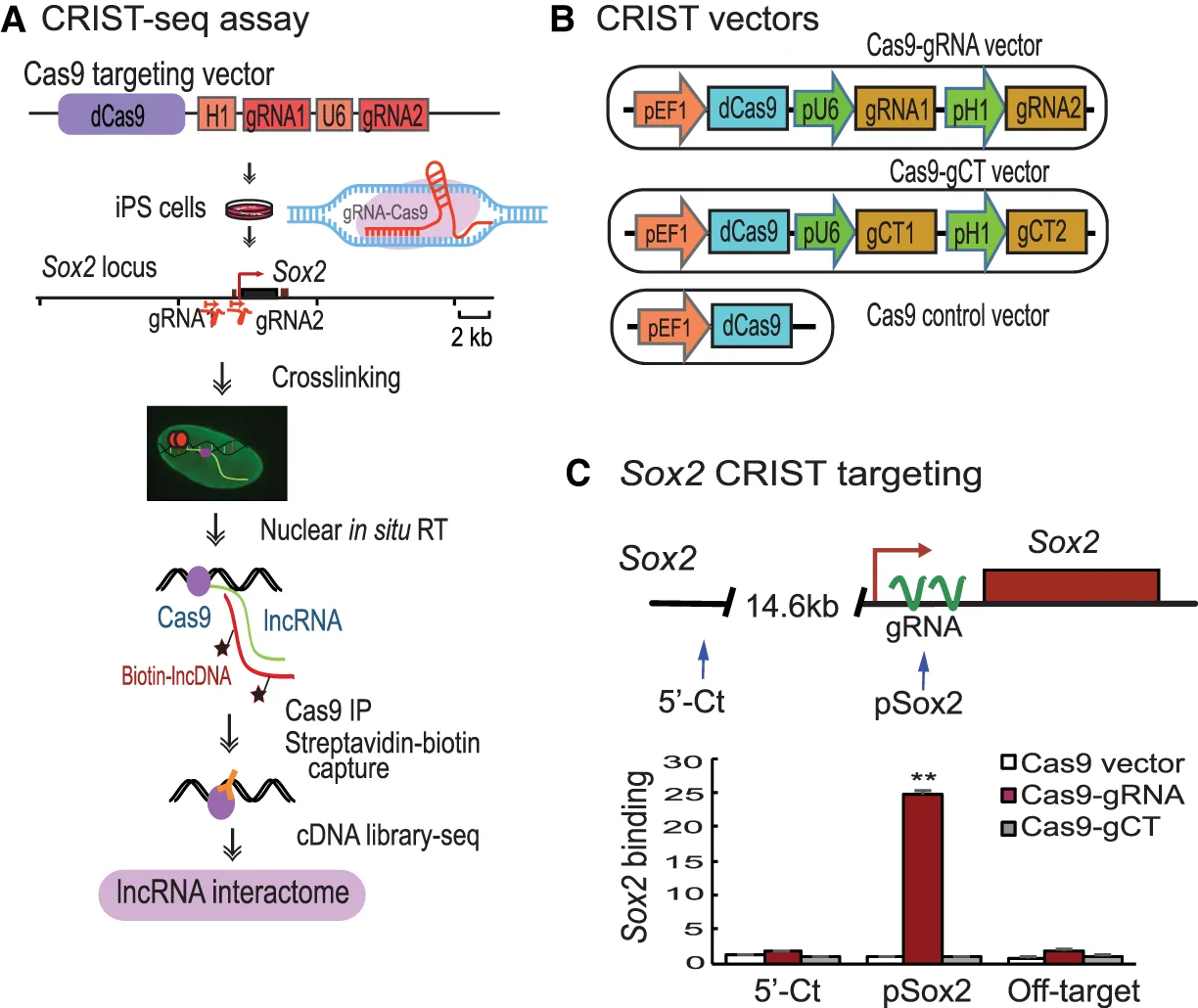
Mapping lncRNA interactions in the *Sox2* promoter by CRIST-seq. (*A*) Schematic diagram of the CRIST-seq assay. (dCas9) The catalytically inactive CRISPR/Cas9; (gRNA) Cas9 guiding RNAs that target the target gene promoter; (pU6) RNA polymerase III *U6* promoter; (pH1) human *H1* RNA polymerase III promoter. Cells were transfected with the Cas9 gRNA cassette that targets the promoter of a given gene. In this study, we targeted the *Sox2* promoter, a well-established core stem cell factor that is required for the maintenance of pluripotency. The Cas9 gRNA-expressing cells were crosslinked by formaldehyde to fix the RNA–DNA structure. After cell membrane lysis, the nuclei were isolated and the promoter-interacting RNAs were in situ reverse transcribed into cDNAs with biotin-dCTP. The promoter biotin-cDNA chromatin complex was immunoprecipitated by an antibody against FLAG, which binds to its target genes through a mechanism of base-pairing between the gRNA and target DNA. After Cas9-FLAG immunoprecipitation, the promoter-interacting biotin-cDNAs were separated from genomic DNAs by streptavidin beads. The CRIST-captured chromatin cDNAs were collected for library construction and sequenced to identify the lncRNAs that interact with the promoter of a target gene. (*B*) CRIST targeting vectors. (gRNA) Cas9 guiding RNAs that target the target gene promoter; (gCT) scrambled control gRNA. The Cas9-gCT vector was used as the CRIST control. The Cas9 vector that lacks the targeting gRNAs was used as the vector control. In the targeting vector, two Cas9 gRNAs are transcribed by human *U6* and *H1* promoters, respectively, and they guide the Cas9 to the promoters of target genes. (*C*) Specific CRIST targeting of the *Sox2* promoter. (pSox2) The targeting site in the *Sox2* promoter where the Cas9 gRNAs are designed; (5′-Ct) a fragment that is 14.6 kb away from the pSox2 target site and is used as the control site. (Cas9 vector) Cells that were treated with the Cas9 control vector that lacks the gRNAs; (Cas9-gRNA) cells that were targeted by both Cas9 and *Sox2* gRNAs; (Cas9-gCT) cells that were treated with the random control gRNA vector. (Off-target) A CRIST control site that is 33.8 kb upstream of the housekeeping gene *GAPDH*. The Cas9 *Sox2*-gRNA iPSCs were fixed with formaldehyde and the chromatin complex was immunoprecipitated with a FLAG antibody and an IgG control antibody (without in situ reverse transcription). Cas9 enrichment signals were quantitated by real-time PCR using specific primers derived from the pSox2 targeting site, 5′-Ct control site, and off-target site. All data shown are mean ± SD from three independent experiments by normalization over the IgG control. (**) *P* < 0.01 as compared with the Cas9 Vector and Cas9-gCT controls. Note the specific enrichment of Cas9 binding at the pSox2 site in Cas9-*Sox2* gRNA targeting group. After confirming the specificity of the Cas9 gRNA, the Cas9 *Sox2*-gRNA iPSCs were then used for CRIST-seq assay.

Nuclear architecture around the regulatory elements is important in the regulation of a target gene. In cellular reprogramming, for example, nuclear architecture undergoes marked remodeling and is coupled with the reactivation of core stem cell factors (Zhang et al. 2013; Hu and Hoffman 2014). We proposed to systematically map lncRNAs that interact with stemness gene promoters in this reprogramming model. Currently, there are no reliable approaches to map the lncRNA network at a specific regulatory element, such as the promoter or enhancer, in a given gene. We therefore devised the CRIST-seq assay to examine lncRNAs that interact with the chromatin complex of stemness gene promoters (Fig. 1A).

The CRIST assay combines the advantage of the simplicity of lncRNA in situ biotin labeling with the specificity of the Cas9 gene editing system. Specifically, cells carrying a catalytically inactive CRISPR/Cas9 and gRNAs were crosslinked to fix the chromatin DNA-RNA structure. After fixation, nuclear in situ reverse transcription was performed to convert the promoter-interacting RNAs into cDNAs in isolated nuclei using biotin-dCTP. The promoter biotin-cDNA complexes were isolated by Cas9-FLAG immunoprecipitation and biotin-streptavidin bead purification. The captured cDNAs were sequenced by Illumina library sequencing to identify the lncRNA components that regulate the activity of a given gene promoter (Fig. 1A; Supplemental Fig. S1).

*Sox2* is a well-established core stem cell factor that is critical for maintaining pluripotency. As a proof-of-concept, we utilized this CRIST assay to map the lncRNAs that interact with the *Sox2* gene promoter complex. To target the *Sox2* promoter, we designed two Cas9 gRNAs from the *Sox2* promoter (Supplemental Fig. S2) and cloned them in a lentiviral vector that carries the catalytically inactive dCas9 (Fig. 1B). The assay control vector was designed to contain a random Cas9 gRNA (gCT). Mouse iPSCs were generated using a *Pou5f1-Sox2-Klf4-Myc* (OSKM) cocktail (Zhang et al. 2013), and pluripotency was characterized by immunohistochemical staining and teratoma assays (Du et al. 2018). The Cas9 gRNA-expressing iPSCs were collected, and the chromatin was crosslinked with formaldehyde to fix the RNA-DNA-Cas9 structure in the promoter. We performed nuclear in situ reverse transcription to convert RNAs into cDNAs using biotin-dCTP. The *Sox2* chromatin biotin-cDNA Cas9 complex was then immunoprecipitated with an anti-FLAG antibody. After reversing the crosslinks, the *Sox2* promoter-associated biotin-cDNAs were separated from genomic DNAs by streptavidin beads, and these cDNAs were used to construct DNA libraries for Illumina sequencing (Supplemental Fig. S1). Using this CRIST-seq approach, we aimed to map the entire lncRNA interacting network in the *Sox2* promoter.

### Specific CRIST targeting of gene promoters

To validate the specificity of the assay, we used quantitative PCR to examine Cas9-gRNA enrichment at the targeting site (pSox2), where the two gRNAs are located, and at the 5′-control site (5′-Ct), which is 14.6 kb away from the pSox2 target site (Fig. 1C, top panel). In the Cas9-gRNA immunoprecipitated chromatin complex, we detected the specific enrichment of *Sox2* promoter DNA (pSox2) (Fig. 1C). No enrichment was detected in the random gRNA control (gCT) or the Cas9 vector control (Vector). Similarly, we did not detect Cas9 enrichment at the 5′-control site (5′-Ct).

To further test the specificity of the assay, we also chose an off-target fragment that is 33.8 kb upstream of the housekeeping gene *GAPDH*. As expected, no Cas9-gRNA enrichment was detected at this off-target control site (Fig. 1C). These data demonstrate the specificity of the CRIST approach to target the *Sox2* promoter.

To test the broad application of this CRIST assay, we designed two gRNAs from the promoter sequence of *Pou5f1* (Supplemental Fig. S3A), a second core stem cell transcription factor that is critical for maintaining pluripotency and for cell reprogramming. After immunoprecipitation, we found enriched Cas9 binding in the *Pou5f1* promoter, whereas very low background signals were detected at the 5′-control site that is about 13.9 kb from the *Pou5f1* promoter and the off-target site (Supplemental Fig. S3B).

We also applied this assay to two human oncogenic factors, proto-oncogene *FLI1* and growth factor *IGF2*, which are aberrantly up-regulated in human tumors. Using specific Cas9 gRNAs, we showed a strong enrichment of the binding signal at the target sites for *FLI1* and *IGF2* (Supplemental Figs. S4, S5). No Cas9 precipitation signals were detected at both the 5′-control sites and the off-target site. Due to the flexibility of gene-specific gRNAs, it is presumed that this CRIST approach can be flexibly applicable to any given regulatory element in the genome.

### CRIST-seq mapping of the lncRNA network in the *Sox2* promoter

*FECR1* is a novel circular RNA derived from exons 4-2-3 of the oncogenic *FLI1* gene. Recently, we showed that this circRNA binds to the *FLI1* promoter, where it recruits TET1 and induces DNA demethylation to activate *FLI1* (Chen et al. 2018). We thus used it as a positive control. Using CRIST-seq, we demonstrated enrichment of *FECR1* circRNA in the *FLI1* promoter (Supplemental Fig. S6). *MALAT1* is also a well-known nuclear lncRNA. However, we did not detect the binding of *MALAT1* to the *FLI1* promoter in the *FECR1*-gRNA CRIST product.

**Figure 2.**
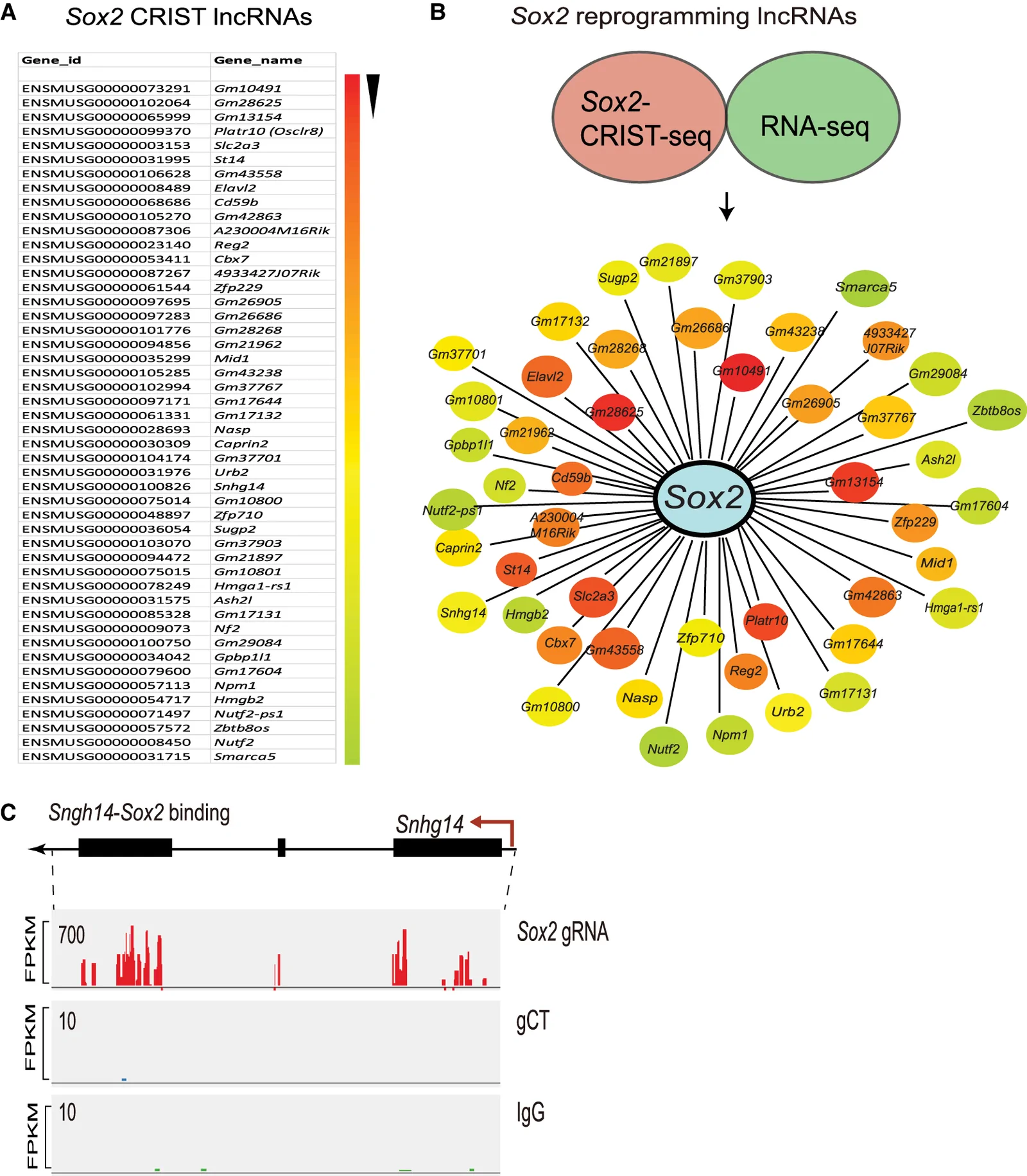
The lncRNA interacting network in the *Sox2* promoter. (*A*) CRIST-seq identifies the top 50 *Sox2* promoter-interacting RNAs. The *Sox2* interacting RNAs are listed in order of the enrichment fold of the top 50 CRIST-seq data. (*B*) The reprogramming-associated *Sox2* lncRNA interacting network. To identify reprogramming-associated lncRNAs, fibroblasts and iPSCs were collected at different stages of reprogramming, and total RNAs were sequenced. Data regarding the RNAs that were changed by greater than twofold were combined with the CRIST-seq data using a VENN program. A cut-off threshold of peak enrichment FPKM > 50 was arbitrarily set to select CRIST-seq RNAs for VENN analysis. Integration of these two data sets generated a total of 59 RNAs, which were differentially expressed in reprogramming and also interacted with the *Sox2* promoter. The *Sox2* RNA interaction was drawn based on the differential expression fold (red to blue) of lncRNAs between iPSCs and fibroblasts. (*C*) Specific binding of reprogramming-associated lncRNA *Snhg14* in the *Sox2* promoter chromatin complex. Three sets of CRIST-seq BAM data (*Sox2*-gRNA, control gCT, and IgG control) were uploaded onto the Integrative Genomics Viewer (IGV) browser (Thorvaldsdóttir et al. 2013), and a Sashimi plot was used to compare the enrichment signal between each group. (FPKM) Fragments per kilobase of exon per million fragments mapped. The CRIST-seq data revealed that all three exons of *Snhg14* interact with the *Sox2* promoter.

We then used CRIST-seq to map the RNA interaction network in the *Sox2* promoter. To define the specific binding of RNAs, the CRIST-seq signal intensities were normalized over that of the nontargeting Cas9 gCT control and the IgG control using parameters of fold change ≥2 and *P*-value < 0.05. The top 50 CRIST-seq RNAs that interact with the *Sox2* promoter are shown in Figure 2A (Supplemental Table S1). The ontology analysis showed that the *Sox2* promoter-interacting RNAs may target multiple pathways that are related to development, metabolism, stem cell maintenance, and differentiation (Supplemental Figs. S7, S8; Supplemental Table S2).

To determine the role of *Sox2*-interacting lncRNAs in reprogramming, we performed conventional RNA-seq for cells collected at different stages of reprogramming, including fibroblasts and iPSCs. By combining the RNA-seq and the *Sox2* CRIST-seq data sets using a VENN program, we identified 59 RNA candidates that not only participate in the formation of the *Sox2* promoter chromatin complex but are also differentially expressed in reprogramming (Fig. 2B; Supplemental Fig. S9). By further combining this data with the *Pou5f1* CRIST-seq data, we identified 27 top RNA candidates that are associated with pluripotency. We were surprised that the *Sox2* pre-mRNA was not among those listed in these top CRIST RNAs. However, using a highly sensitive qPCR assay, we were able to detect *Sox2* pre-mRNA in the CRIST-seq library product (Supplemental Fig. S10), suggesting that the signal of *Sox2* pre-mRNA in the CRIST complex is relatively weak as compared with that of other *Sox2*-interacting RNAs.

The CRIST-seq assay identified two *Sox2* promoter-interacting lncRNAs, NONMMUT043505 (*Platr10* as named by RNA-seq [Bergmann et al. 2015], *Spilr9*: *Sox2* promoter interacting lncRNA 9), and ENSMUSG00000100826 (*Snhg14*, *Spilr14*: *Sox2* promoter interacting lncRNA 14). The CRIST-seq Integrative Genomics Viewer (IGV) (Thorvaldsdóttir et al. 2013) analysis showed strong interaction signals of *Snhg14* with the *Sox2* promoter in cells carrying the Cas9-*Sox2* gRNA (Fig. 2C). As expected, no lncRNA binding signals were detected in cells carrying the Cas9 random control (gCT, middle panel) and in the IgG immunoprecipitation control (IgG, bottom panels). The RNA-seq IGV data also showed that *Snhg14* was differentially expressed in reprogramming; there was abundant expression in iPSCs, but almost no expression in fibroblasts (Supplemental Fig. S11). RNA-DNA FISH also showed colocalization of *Snhg14* lncRNA in the *Sox2* locus (Supplemental Fig. S12).

We used a RAT-seq approach (Sun et al. 2014; Wang et al. 2014; Du et al. 2018) to validate this lncRNA-DNA interaction. In this assay, lncRNA *Snhg14* was labeled by biotin-dCTP and was pulled down by biotin-streptavidin beads. The pulled-down chromatin DNA complex was used for qPCR. Using this approach, we confirmed the binding of *Snhg14* lncRNA at the *Sox2* promoter. No binding was detected at the 5′-Ct, the downstream D and E sites, and the 3′-Ct control site (Supplemental Fig. S13).

### The *Sox2* promoter-interacting lncRNAs are associated with reprogramming

**Figure 3.**
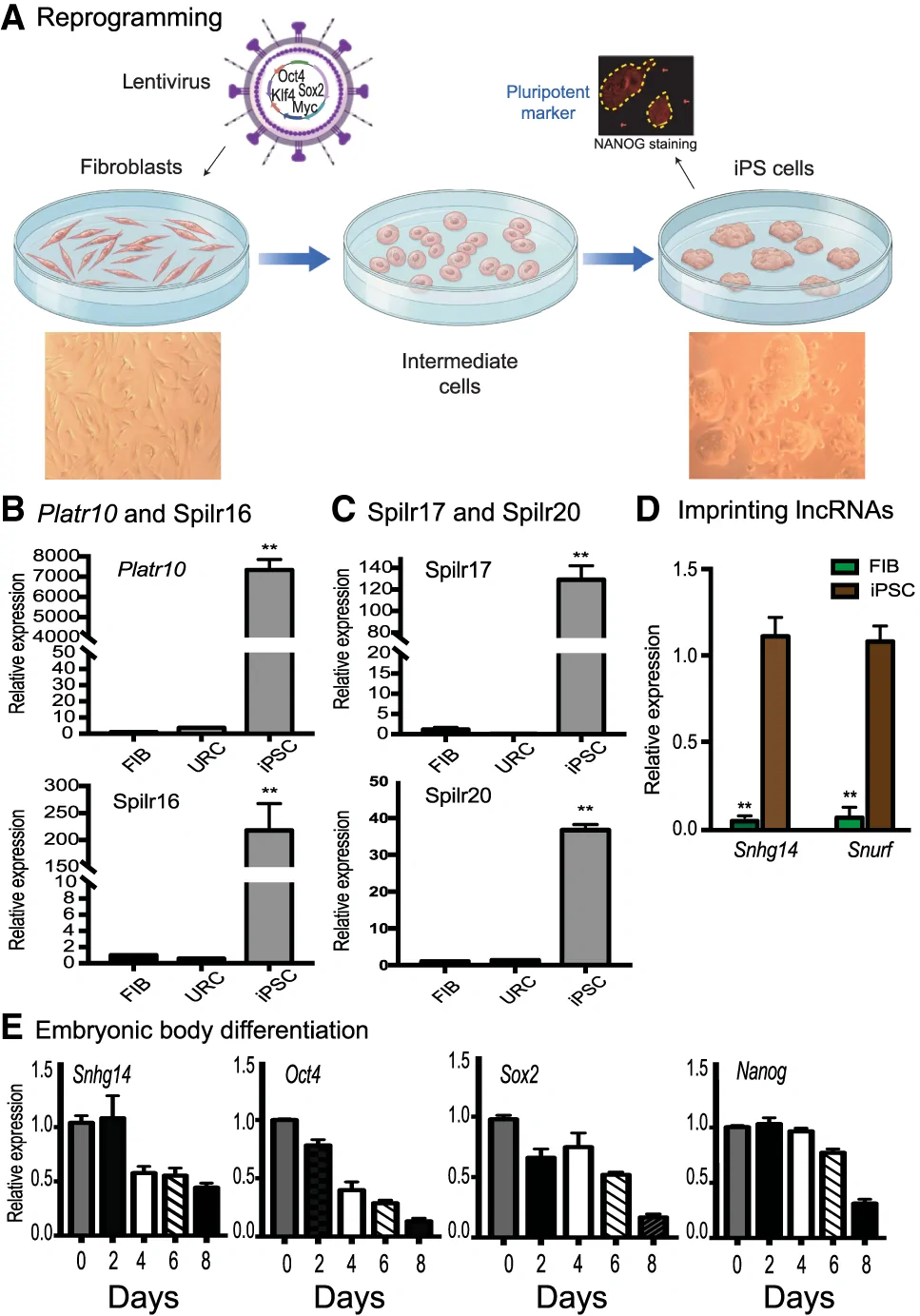
*Sox2*-interacting lncRNAs are associated with reprogramming. (*A*) Mouse fibroblasts were reprogrammed into iPS cells by lentiviral transduction of OSKM (*Oct4*, *Sox2*, *Klf4*, *c-Myc*) factors. After reprogramming, iPSC colonies acquired pluripotency-associated characteristics and were stained positive for pluripotency marker (NANOG). *Bottom* panels: Images of fibroblasts and iPSCs were collected during reprogramming. (*B*) Differential expression of the *Sox2*-binding lncRNAs (*Platr10* and *Spilr16*) in reprogramming. (Fib) Fibroblasts; (URC) unreprogrammed cells that express the OSKM factors, but fail to complete reprogramming; (iPSC) induced pluripotent stem cells; (Spilr) *Sox2* promoter-interacting long noncoding RNA. The data shown are mean ± SD from three independent experiments. (**) *P* < 0.01 as compared with fibroblasts and unreprogrammed cells. (*C*) Differential expression of *Spilr17* and *Spilr20*. (**) *P* < 0.01 as compared with fibroblasts and unreprogrammed cells. (*D*) Quantitative PCR of imprinted *Snurf* and *Snhg14* in reprogramming. (**) *P* < 0.01 as compared with iPSCs. (*E*) Dynamic expression of *Snhg14* in embryoid body differentiation. iPSCs were collected at different stages of embryoid body formation and used for quantitative PCR. Note the similar expression pattern of *Snhg14* to the stem cell marker genes.

It is critical to determine if these *Sox2*-lncRNAs are functionally associated with reprogramming. We collected cells at different stages of reprogramming, including fibroblasts and iPSCs, and examined the expression of the *Sox2* promoter-interacting lncRNAs (Fig. 3A). We then used quantitative PCR to examine the abundance of several *Sox2* promoter-interacting lncRNAs (Spilr) from *Sox2*-*Snhg14* CRIST-seq/RNA-seq candidates. We found that the expression of these Spilr lncRNAs was closely correlated with the status of pluripotency. They were silenced in fibroblasts but became activated in iPSCs during reprogramming (Fig. 3B,C).

Among the RNAs that interacted with the *Sox2* promoter, two were derived from well-known imprinted genes located in the locus related to the Prader-Willi syndrome. *Snurf* is expressed from the paternal allele and can encode the small nuclear ribonucleoprotein N (*Snrpn*) from a downstream open reading frame. *Snhg14* is a paternally imprinted RNA that is thought to share a promoter and exons with the *Snrpn* and *Snurf* genes. The CRIST-seq data showed that these two imprinted RNAs interacted with the *Sox2* promoter. Using quantitative PCR, we found that both were also differentially expressed in cells collected at different stages of reprogramming, with abundant expression in iPSCs and embryonic stem cells as compared with fibroblasts (Fig. 3D).

We also collected cells during the process of embryoid body differentiation from iPSCs. Using quantitative PCR, we found that *Snhg14* became significantly down-regulated during embryoid body differentiation. Its expression pattern was similar to those of core stem cell factor genes *Pou5f1*, *Sox2*, and *Nanog* (Fig. 3E). Collectively, these data suggest that the *Sox2* promoter-interacting lncRNAs are associated with reprogramming.

### The *Sox2*-interacting lncRNAs maintain optimal expression of core stem cell factors

**Figure 4.**
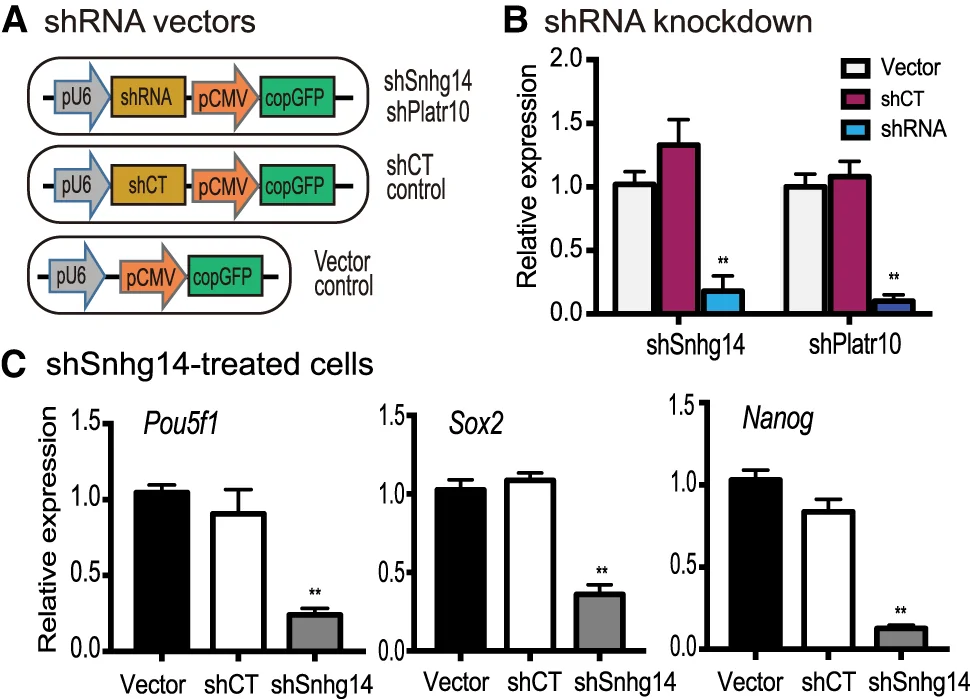
Knockdown of *Sox2*-interacting lncRNAs down-regulates stemness genes. (*A*) Lentiviral shRNA knockdown vectors. (pU6) RNA polymerase III *U6* promoter; (pCMV) CMV promoter; (CopGFP) fluorescence tracking marker; (shRNA) shRNAs targeting *Snhg14* and *Platr10*; (shCT) shRNA random control. Lentiviruses were packaged in 293T cells and were used to infect iPSCs. (*B*) Knockdown of *Snhg14* and *Platr10* lncRNAs in iPSCs. After lentiviral transfection, the shRNA-expressing iPSCs were selected by puromycin and were collected for quantitative PCR. (**) *P* < 0.01 as compared with shCT and vector controls. (*C*) Down-regulation of three core stem cell factor genes in *Snhg14*-knocking down iPSCs. (**) *P* < 0.01 as compared with shCT and vector controls.

To further address the role of these lncRNAs, we used lentiviral shRNAs to knock down the *Sox2*-interacting lncRNAs (Fig. 4A), using *Snhg14* (*Spilr14*) as an example. Lentiviruses carrying the empty lentiviral vector (Vector) and the random shRNA (shCT) were used as the controls. The CopGFP reporter protein in the shRNA lentivirus was used to track lncRNA knockdown iPSCs. Using quantitative PCR, we showed that both lncRNAs were significantly knocked down by shRNAs in iPSCs (Fig. 4B).

After knockdown of *Snhg14*, we found that three core stem cell factor genes, *Pou5f1*, *Sox2*, and *Nanog*, became significantly down-regulated in iPSCs (Fig. 4C, *P* < 0.01). As a control, transfection with lentiviruses carrying the random control shRNA (shCT) and the vector (Vector) did not affect the expression of these stem cell factors. These data suggest that the *Sox2*-interacting lncRNAs are critical for the maintenance of optimal activity of the three core stem cell factor genes in iPSCs.

### The *Sox2*-interacting lncRNAs are required for maintenance of pluripotency

**Figure 5.**
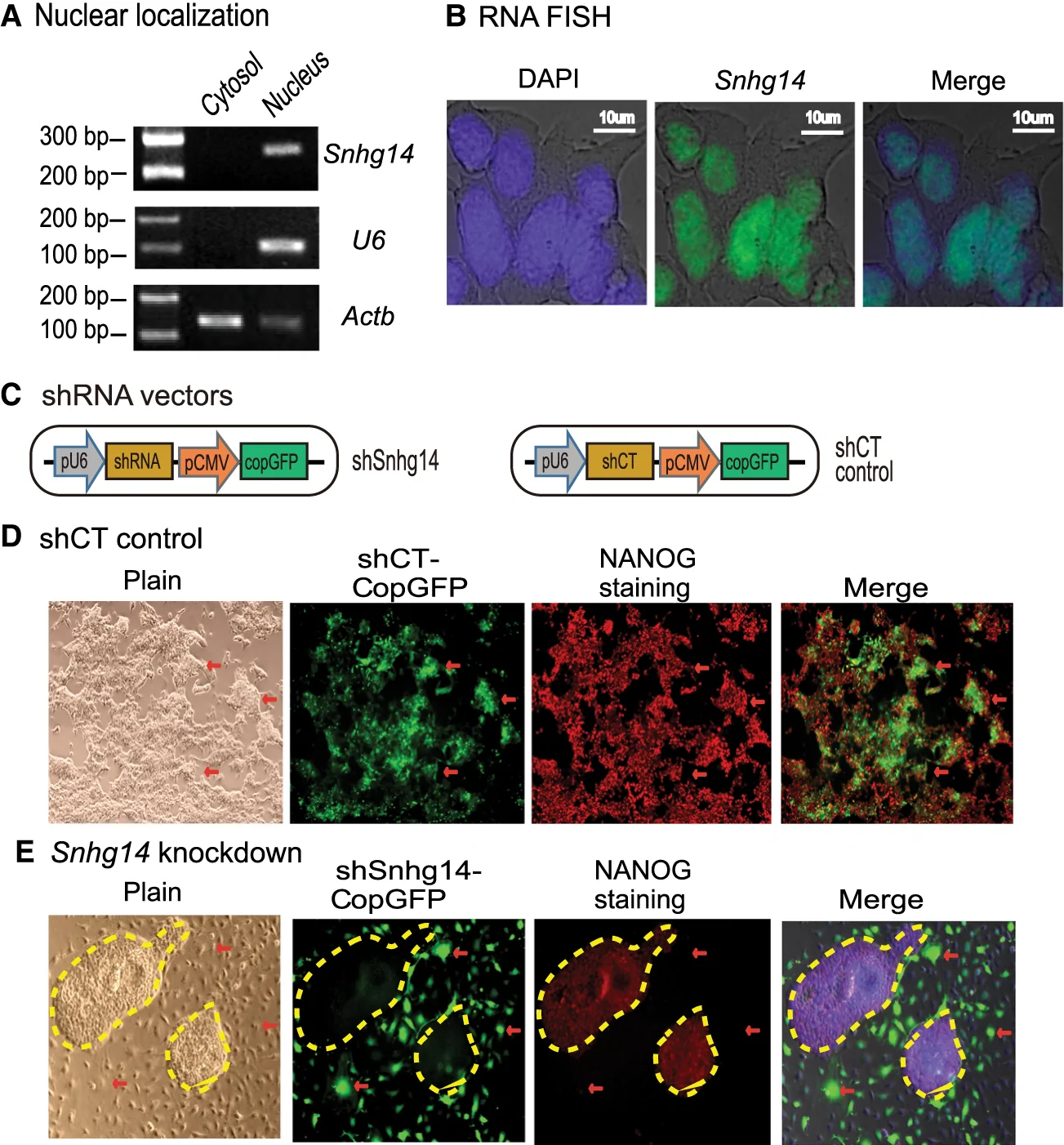
*Sox2*-interacting lncRNAs are important in maintaining pluripotency. (*A*) Subcellular localization of *Snhg14* lncRNA. Cytoplasmic and nuclear RNAs were isolated, and RT-PCR was used to quantitate the subcellular distribution of *Snhg14*. *Actb* was used as the cytoplasmic control and *U6* was used as the nuclear control. (*B*) RNA-FISH of *Snhg14*. To detect the mature RNA, the DIG-11-dUTP probe was synthesized as a short single-strand DNA to cover the intron splicing site. The *Snhg14* signal was detected by anti-digoxigenin-fluorescein (green). DAPI was used to stain the nucleus of iPSC (blue). *Snhg14* was predominantly located in the nucleus. (*C*) The shRNA knockdown vectors. The CopGFP in the lentiviral vector was used as the marker to track the shRNA knockdown cells. (*D*) The iPSCs transfected with shRNA control (shCT). Pluripotency was examined by immunohistochemical staining of critical stem cell marker NANOG protein. The lentivirus-transfected iPSCs expressed the CopGFP fluorescence marker (red arrow) and still stained positive for the pluripotency marker NANOG (red). These cells maintained the original cell morphology. (*E*) The *Snhg14* knockdown iPSCs. (Red arrow) The shRNA-knocked down cells that show the exit from pluripotency, with enlarged and flat cell morphology. The *Snhg14* knockdown iPSCs lost the NANOG pluripotency marker. The yellow-marked areas represent the “island” cells that escaped lentiviral infection. They maintained the compact stem cell appearance and stained positive for the NANOG marker.

Using a subcellular fractionation assay, we showed that *Snhg14* was primarily located in the nucleus (Fig. 5A). The nuclear localization of *Snhg14* was also validated by RNA-FISH using a short DNA probe that covers the intron splicing site to probe the mature RNAs (Fig. 5B).

We then examined if the knockdown of *Snhg14* affects pluripotency of iPSCs. The CopGFP marker in lentiviral vectors was used to track the lncRNA knockdown cells (Fig. 5C). In iPSCs that were transfected with the random control shRNA (shCT), we found that the infected cells were CopGFP-positive and maintained the same cell morphology as pluripotent stem cells (Fig. 5D, panels 1, 2). However, knockdown of lncRNA *Snhg14* altered cell morphology (Fig. 5E, panels 1, 2, red arrows). These lncRNA-knockdown cells became enlarged and flat, appearing like fibroblasts. In the shRNA-treated group, some “island” cells escaped lentiviral infection and did not express the CopGFP tack marker. They still reserved the original compact shape of iPSCs (yellow marked areas without CopGFP fluorescence).

We further examined the pluripotency of the treated iPSCs by performing immunohistochemical staining for the pluripotency-associated marker protein NANOG. As expected, the shCT control group showed extensive expression of NANOG in iPSCs (Fig. 5D, panel 3). After shRNA knockdown of the *Sox2*-interacting lncRNAs, iPSCs became differentiated and lost the pluripotency-associated marker NANOG (Fig. 5E, panel 3, unmarked regions, red arrow). Thus, knockdown of these *Sox2*-interacting lncRNAs caused loss of pluripotency. It should be noted that the relatively low CopGFP fluorescence in the shCT group was associated with weaker activity of the CMV promoter in iPSCs than that seen in differentiated cells in shRNA-treated groups.

### *Sox2*-interacting lncRNA *Snhg14* activates stem cell factor gene promoters

**Figure 6.**
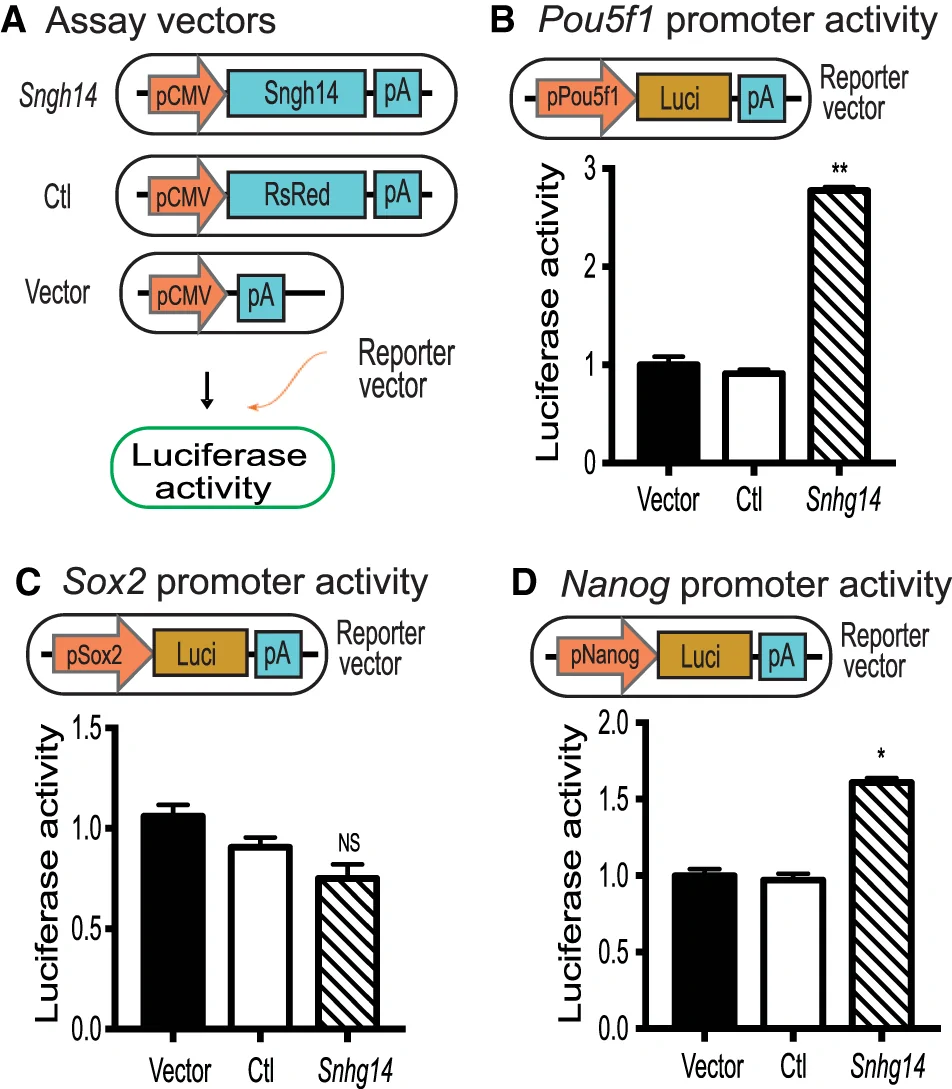
*Snhg14* lncRNA activates stem cell core factor gene promoters. (*A*) LncRNA expression vectors. *Snhg14* was expressed under the control of the CMV promoter. RsRed fluorescence marker was used as the expression control (Ctl), and the empty vector was used as the vector control (Vector). The expression vectors were cotransfected with stem cell core factor gene promoter-luciferase reporter vector in 293T cells and luciferase activity was measured. (*B*) *Snhg14* activates the *Pou5f1* promoter. (pPou5f1) *Pou5f1* promoter; (Luci) luciferase; (pA) SV40 poly(A) signal. A 4-kb *Pou5f1* promoter DNA fragment was cloned in front of luciferase. 293T cells were cotransfected with *Snhg14* expression vector and the pPou5f1-luciferase vector DNAs using Lipofectamine 2000. Forty-eight hours after transfection, luciferase activity was measured using a Promega luciferase assay kit. The activity of luciferase was adjusted by using the Vector control as 1. (**) *P* < 0.01 as compared with the Ctl and Vector controls. (*C*) The activity of the *Sox2*-luciferase. The *Snhg14* expression vector DNA was cotransfected with the pSox2-luciferase vector DNA in 293T cells. (NS) No statistical significance as compared with the Ctl and Vector controls. (*D*) Effect of *Snhg14* on the activity of the *Nanog* promoter. The *Snhg14* expression vector DNA was cotransfected with the pNanog-luciferase vector DNA in 293T cells. (*) *P* < 0.05, (**) *P* < 0.01 as compared with the Ctl and Vector controls.

To further characterize the role of *Sox2* promoter-interacting lncRNAs, we cloned lncRNA *Snhg14* into a pCMV-RsRed-Puro vector to determine if it affected the activity of core stem cell factor gene promoters in a luciferase reporter assay (Fig. 6A). The empty vector and the RsRed expression vector were used as the assay controls. By cotransfecting the lncRNA-overexpressing vectors with stemness gene promoter-luciferase reporter vectors in 293T cells, we found that *Snhg14* enhanced the activity of the *Pou5f1* and *Nanog* promoters but had less effect on the *Sox2* promoter (Fig. 6B–D).

### LncRNA *Snhg14* enhances pluripotent reprogramming

**Figure 7.**
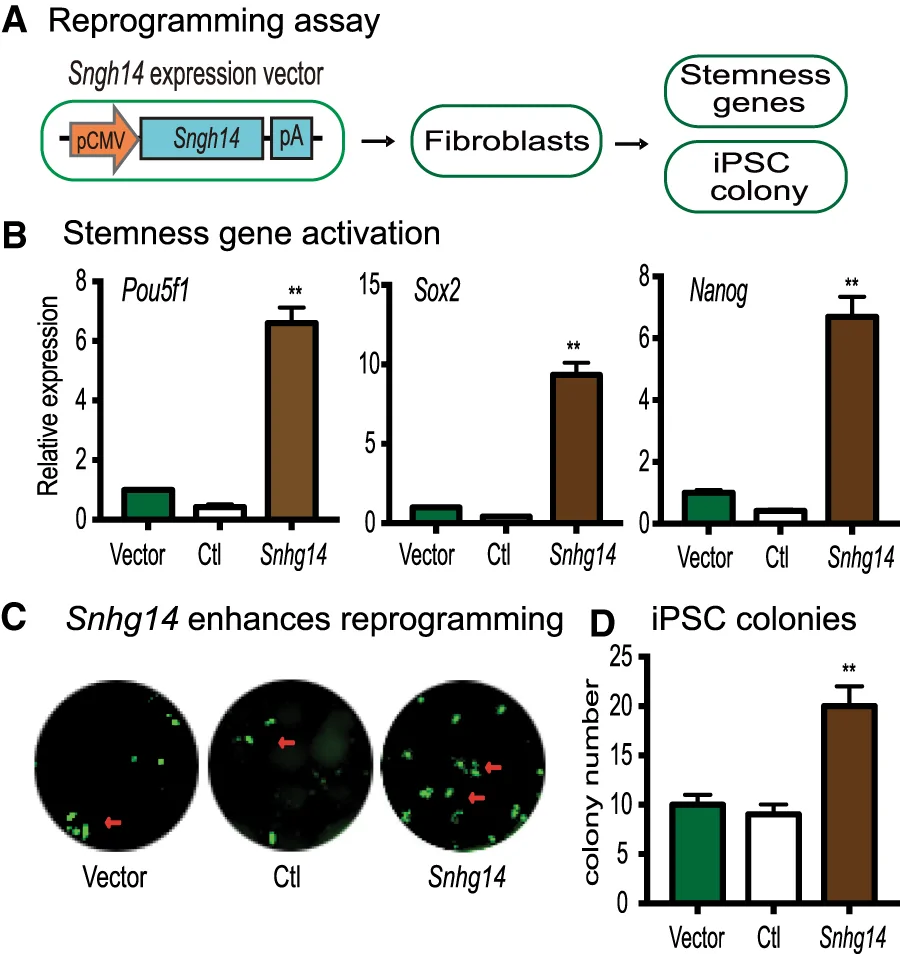
*Snhg14* lncRNA enhances pluripotent reprogramming. (*A*) Schematic diagram of the reprogramming assay. Fibroblasts and MEF cells were transfected with *Snhg14* lentivirus. After puromycin selection, the *Snhg14*-expressing cells were collected for quantitative PCR measurement of the endogenous stemness genes (*Pou5f1*, *Sox2*, and *Nanog*) or for reprogramming. (*B*) Activation of the endogenous stemness genes by *Snhg14*. Expression of *Pou5f1*, *Sox2*, and *Nanog* was measured by quantitative PCR and calculated as relative expression by setting the Vector control as 1. (**) *P* < 0.01 as compared with the Ctl and Vector controls. (*C*) *Snhg14* enhances the efficiency of reprogramming. MEF cells were transfected with the lentiviruses carrying the empty vector (Vector), lncRNA control (Ctl), and *Snhg14*. After doxycycline (DOX) induction, the iPSC colonies were immunostained using anti-NANOG antibody (green). (*D*) Quantitation of iPSC colonies. iPSC colonies per well were counted and averaged from three independent assays. (**) *P* < 0.01 as compared with the Ctl and Vector controls.

After confirming the role of *Snhg14* on stem cell factor gene promoters, we were then interested in learning if this lncRNA affected pluripotent reprogramming. We first used a lentiviral vector to stably express *Snhg14* in mouse fibroblasts (Fig. 7A). For control groups, cells were transfected with either the empty lentiviral vector or the RsRed fluorescent marker. After selection with puromycin, cells were collected and total RNA was extracted to examine the expression of endogenous stem cell factor genes. As seen in Figure 7B, ectopic expression of *Snhg14* activated these endogenous stem cell factor genes. In control groups treated with the empty vector (Vector) or RsRed (Ctl), there was no activation of these factor genes.

Given the fact that the activation of these three core stem cell factors is a critical event in pluripotent reprogramming, we used a doxycycline-inducible system (Zhuang et al. 2018) to examine if *Snhg14* had a similar impact on the reprogramming process. OG2 MEF cells were first transfected with the *Snhg14* and control lentiviruses. After puromycin selection, cells were switched to the reprogramming media containing 2 µg/mL doxycycline (DOX). After induction, iPSC colonies were stained for the pluripotent marker NANOG. Compared with the vector (Vector) and the RsRed (Ctl) controls, ectopic expression of *Snhg14* significantly enhanced reprogramming of MEF cells into pluripotency (Fig. 7C,D).

## Discussion

Chromosomes and individual genes occupy preferred locations within the nucleus and form dynamic three-dimensional interactions as intra- and interchromosomal loops and bridges. LncRNAs may be important regulatory components of these chromatin interactions. Chromatin-interacting lncRNAs may function in *cis* and in *trans* to regulate the transcriptional activity of functional genes. However, the mechanisms by which the lncRNAs govern spatial chromatin positioning in stem cells are not well understood. In this study, we have developed a CRIST-seq approach to map lncRNA interactions in the *Sox2* promoter. This assay combines the simplicity of lncRNA in situ labeling with the specificity of the Cas9 gene editing system. Using flexibly designed gRNAs to target genes, we mapped the lncRNA interacting network in the promoter of pluripotency-associated marker gene *Sox2*. Using this approach, we demonstrate a critical role of the *Sox2* promoter-interacting lncRNAs during reprogramming and describe a physiologically important, pluripotency-specific lncRNA network in the *Sox2* promoter.

In this study, we showed that multiple RNAs constitute critical components of the *Sox2* promoter complex. These *Sox2*-interacting RNAs were critical for the maintenance of pluripotency, being silenced in fibroblasts, activated in pluripotent reprogramming, and down-regulated following differentiation. Knockdown of the *Sox2*-interacting RNAs caused the exit of stem cells from pluripotency. On the other hand, lentiviral expression of the lncRNAs activated core stem cell factor genes and enhanced fibroblast reprogramming into pluripotency. Taken together, our data suggest that lncRNAs may function as cofactors to participate in the organization of a pluripotent chromatin network of core stem cell factor genes (Zhang et al. 2013; Hu and Hoffman 2014).

Our CRIST-seq data suggest a new model for the function of the chromatin lncRNAs in the initiation of pluripotency. During reprogramming, pluripotency-associated lncRNAs are transcribed and act in concert with other chromatin factors to coordinate the formation of a pluripotency-specific topological architecture and activate core stem cell core factor genes, like *Sox2* and *Pou5f1*. Coordinated expression of these stem cell core factors synergizes to promote reprogramming of somatic cells into pluripotency. In unreprogrammed fibroblasts, however, the genes encoding the pluripotency-associated lncRNAs are not expressed. Without appropriate expression of the appropriate stem cell factors, reprogramming cannot be fully initiated.

It should be emphasized that the CRIST-seq assay just maps the RNAs that interact with the promoter DNA. The lncRNAs that are identified may interact with the promoter DNA directly or indirectly. In addition, a lncRNA may bind to more than one gene target. For example, *Platr10* and *Snhg14* bind to both the *Sox2* and *Pou5f1* promoters. Further studies are needed to address whether these RNAs physically interact with *Sox2* and whether these *Sox2* DNA targets are also colocalized as essential factors for reprogramming. Furthermore, functional assays may be performed to validate these interactions. We will need to determine the specific promoter-binding elements in the lncRNA molecule to learn if the deletion of this element in the lncRNA abolishes the function of the lncRNA. Finally, it is interesting to note that ectopic expression of *Snhg14* also significantly up-regulates the *Nanog* gene, even though the CRIST-seq assay did not reveal this interaction. It is possible that this lncRNA is able to activate the pluripotent network, which includes the *Nanog* gene. Alternatively, the activated *Pou5f1* and *Sox2* may themselves activate other stemness genes, like *Nanog*. Further studies are needed to explore the underlying mechanism.

These CRIST-seq data also demonstrate that, in addition to lncRNAs, there are some coding mRNAs that interact with the *Sox2* promoter chromatin complex (Supplemental Table S1). Pathway analysis shows that these coding mRNAs are involved in the cAMP signal pathway, adherens junction, phospholipase D signaling pathway, glutamatergic synapse, and lipolysis (Supplemental Figs. S3, S4). In a separate study, using both the cellular compartment fraction assay and RNA-FISH assays, we confirmed the presence of the *Sox2* promoter interacting mRNA *Snurf* in both the cytoplasm and the nucleus. The qPCR primers were designed to cover the intron splicing site. Thus, the RNAs detected should be the mature mRNAs, and not pre-mRNAs. It is not clear why and how these coding mRNAs participate in the formation of the *Sox2*-promoter interacting network. Perhaps these coding mRNAs have dual functions during the process of reprogramming. In the cytoplasm, they function as messenger RNAs, where they are translated into proteins. In the nucleus, however, these mRNAs may function in a structural manner similar to that of the long noncoding RNAs. By binding to the *Sox2* promoter, they may be directly involved in the regulation of the stem cell genes. Future studies are needed to exclude the possibility of a CRIST-seq artifact and to address the functions of these coding mRNAs in reprogramming.

In summary, we have devised a CRIST-seq approach to broadly profile the lncRNA-DNA interacting network at a given genomic locus. Using this technique, we have uncovered a unique lncRNA interaction profile in the *Sox2* promoter. The *Sox2* promoter-interacting lncRNAs are critical for the maintenance of pluripotency. Understanding this pluripotency-specific topological lncRNA-DNA interacting network may reveal valuable insights into how a gene and its associated lncRNAs act in concert to control cell fate during reprogramming and lineage differentiation. Additionally, this CRIST approach can be used to map lncRNA interactions in other key factor genes simply by replacing the gene-specific gRNAs in the assay. Thus, this CRIST-seq technology can be broadly used to screen lncRNAs and mRNAs that interact with any chromatin regulatory regions, such as promoters or enhancers.

## Methods

### Cell lines and cell culture

Mouse muscle-derived fibroblasts cultured from a 129 mouse fetus were used for this study (Zhai et al. 2015). Briefly, fresh fetal tissues were minced into small pieces and cultured in six-well plates with minimum DMEM medium to cover the tissue. After 4–6 d, fibroblast-like cells around the tissue were digested with trypsin. Fibroblasts were maintained in DMEM (Sigma-Aldrich) containing 10% (v/v) fetal bovine serum (Sigma-Aldrich), 1% (v/v) of penicillin-streptomycin (Sigma-Aldrich) at 37°C in 5% CO_2_ air atmosphere.

### Pluripotent reprogramming

Mouse fibroblasts were reprogrammed into induced pluripotent stem cells (iPSCs) by *Pou5f1*-*Sox2*-*Klf4-Myc* (OSKM) cocktail factors (Chen et al. 2012, 2016; Zhang et al. 2013; Zhai et al. 2015). Briefly, the OSKM lentiviruses were packaged in 293T cells by cotransfecting the lenti-OSKM with viral packaging vectors using Lipofectamine 2000 (Invitrogen). The virus-containing supernatants were concentrated with Amicon Ultra-15 Centrifugal Filter Units (Millipore). Fibroblasts were infected with concentrated lentiviruses in the presence of polybrene (8 µg/mL). After infection, the cells were transferred to 100-mm dishes on mitomycin C-inactivated MEF feeder cells. The iPSC clones were maintained in KnockOut DMEM (Gibco) containing 15% (v/v) KnockOut SR (Gibco), 1% (v/v) of penicillin-streptomycin (Sigma-Aldrich), 2 mM L-Glutamine solution (Sigma-Aldrich), 1× MEM-NEAA (Invitrogen), 200 µM 2-Mercaptoethanol (Sigma-Aldrich), and 1000 U/mL LIF (Millipore) at 37°C in 5% CO_2_ air atmosphere. The unreprogrammed fibroblasts and iPSCs were collected for subsequent experiments. The iPSCs were expanded and characterized by examining the expression of pluripotent markers, NANOG and FUT4 (also known as SSEA1), and by the teratoma assay using the method as previously described (Chen et al. 2012; Zhang et al. 2013).

### Mapping of the promoter lncRNA interacting network by CRIST sequencing

A chromatin-RNA in situ reverse transcription sequencing (CRIST-seq) assay was devised to map the promoter-interacting lncRNAs. The promoter-interacting RNAs were in situ reverse transcribed into cDNAs with biotin-dCTP. The biotin-cDNA chromatin complexes were immunoprecipitated with a FLAG antibody and were purified by streptavidin beads for library sequencing (Supplemental Fig. S1).

We constructed the Cas9-*Sox2* gRNA vector by cloning two *Sox2* promoter gRNAs into the lenti Cas9-*IGF2* gRNA vector that contains the catalytically inactive Cas9 (dCAs9) (Zhang et al. 2017). The pU6-gRNA1-pH1-gRNA2 cassette was synthesized by joining the *H1* promoter with two oligonucleotides that contain the guiding RNA (gRNA) from the *Sox2* promoter, including *Sox2*-gRNA1: 5′-GGGGTTGAGGACACGTGCTG-3′ and *Sox2*-gRNA2: 5′-GAGCCAATATTCCGTAGCAT-3′, respectively (Supplemental Fig. S2; Supplemental Table S3). The expression cassette was inserted downstream from the *U6* promoter in the vector using PmeI and NotI (Zhang et al. 2017). The Cas9 control vectors were constructed by replacing target gRNAs with two scrambled guiding RNAs: gCT1: 5′-GTTCCCTGCAAGAGTGCCCA-3′ and gCT2: 5′-GCACTACCAGAGCTAACTCA -3′).

The Cas9-*Sox2* gRNA lentiviruses were produced in 293T cells as previously described (Zhai et al. 2015; Chen et al. 2016; Zhang et al. 2017). The viral supernatants were filtered with a 0.45-μm filter, concentrated by a PEG-it kit (SBI), aliquoted, and stored at −80°C. An aliquot of the Cas9-*Sox2* gRNA lentivirus was used to transfect mouse iPSCs and fibroblasts. After transfection, cells were selected by puromycin and collected for immunoprecipitation.

As an initial step of the assay, we performed an immunoprecipitation to assess its specificity for the Cas9 *Sox2* gRNA. Cells carrying the dCas9 *Sox2*-gRNA, dCas9-gCT, and dCas9 cassettes were crosslinked with 2% formaldehyde and lysed with cell lysis buffer (10 mM Tris [pH 8.0], 10 mM NaCl, 0.2% NP-40, 1× protease inhibitors). Conventional chromatin immunoprecipitation (ChIP) was performed using an anti-FLAG antibody (F1804, Sigma-Aldrich). An anti-IgG antibody (ab171870, Abcam) was used as the background control for ChIP. As described, qPCR was used to map the binding specificity of Cas9 *Sox2*-gRNA in the gene locus and other “off-target” loci.

After confirming the specific binding of Cas9 *Sox2*-gRNA, we then performed the CRIST-seq assay to map the *Sox2*-interacting lncRNAs. Nuclei were collected, suspended in 1× reverse transcription buffer in the presence of 0.3% sodium dodecyl sulfate (SDS), and incubated at 37°C for 1 h. Triton X-100 was then added to a final concentration of 1.8% to sequester the SDS. DNA from an aliquot of nuclei (3 × 10^6^) was reverse transcribed with Maxima Reverse Transcriptase (Thermo Fisher Scientific) at 37°C for 30 min in a 20-µL reaction with biotin-dCTP (1 µL random hexamer, 1 µL 10 mM dNTP, 1 µL 0.4 mM biotin-dCTP, 1 µL RT enzyme, 0.5 µL RNase inhibitors, 1 µL 0.1 M DTT, 4 µL 5× cDNA synthesis buffer, RNase-free water to 20 µL). The reaction was stopped by adding 4 µL 0.5 M EDTA. After nuclear lysis, the biotin-cDNA/chromatin DNA complex was subjected to sonication and was immunoprecipitated with an anti-FLAG antibody (F1804, Sigma-Aldrich). After reversing the crosslinks, *Sox2* promoter-interacting biotin-cDNAs were purified from genomic DNAs using M-280 streptavidin beads (Invitrogen). The second strand cDNA was synthesized using a Stratagene cDNA Synthesis kit (Agilent Technologies). The double-stranded cDNAs were digested by DpnI and were used for library construction by ligating with the NEBNext adaptors (NEBNext ChIP-seq Library Prep Master Mix Set for Illumina). The cDNA library was subjected to Illumina sequencing (Shanghai Biotechnology) as described in the above section. The gRNA sequences for these target genes and their off-target control gRNAs are listed in Supplemental Table S3. For CRIST-seq control, we performed the CRIST assay using random gRNAs (gCT) and constructed the control library for sequencing using the same protocol. At the same time, an anti-IgG antibody was used as the background control for immunoprecipitation. These two libraries were sequenced in parallel with Cas9-*Sox2* gRNA samples.

### CRIST-seq data analysis

As previously described (Du et al. 2018), the raw data and the low-quality data were filtered using FASTX software (v0.0.13; http://hannonlab.cshl.edu/fastx_toolkit/). Clean reads were mapped to the mouse mm10 genome by TopHat software (version 2.0.9) (Trapnell et al. 2009). The mapped RNA reads were quantitated as “fragments per kilobase of transcript per million fragments mapped” (FPKM) using Cufflinks (version 2.1.1) (Trapnell et al. 2010). BedGraph files were visualized in UCSC Genome Browser (https://genome.ucsc.edu). The peak was called and annotated with RIPSeeker (Abdelmohsen et al. 2013) and was adjusted over the peaks overlapping with the IgG control enriched regions. The CRIST-seq signal intensities were further normalized over that of the nontargeting Cas9 gCT control using the DiffBind package (Ross-Innes et al. 2012) (fold change difference ≥ 2 and *P*-value < 0.05, with false discovery rate [FDR] < 0.1). The normalized sequencing data were used to map the *Sox2* RNA interaction network.

### RNA-seq to identify differentially expressed lncRNAs in reprogramming

We proposed to combine CRIST-seq data with RNA-seq data to identify lncRNAs that not only interact with the *Sox2* promoter but are also differentially expressed in pluripotent reprogramming. For RNA-seq, total RNA was isolated from fibroblasts and iPSCs collected in reprogramming using TRIzol reagent (Invitrogen) (Zhang et al. 2013; Zhai et al. 2015). The indexed libraries were prepared using Illumina's TruSeq RNA Sample Prep kit and were paired-end sequenced by Shanghai Biotechnology (Shanghai). The clean reads were mapped to the mouse genome (genome version: mm10, GRCm38.p4) using the STAR software (Dobin et al. 2013). Cuffdiff (Trapnell et al. 2013) was used to calculate the differentially expressed RNAs using fold change > 2 and *P* < 0.05 with an unpaired two-sided *t*-test.

To identify reprogramming-associated lncRNAs, a VENN program (http://bioinformatics.psb.ugent.be/webtools/Venn/) was used to integrate the RNA-seq RNAs (greater than twofold and *P* < 0.05) with the CRIST-seq RNAs (peak enrichment FPKM > 50 as a cut-off threshold after adjusting over the IgG control and Cas9-gCT control). The overlapping RNAs identified by these two data sets were chosen for further function characterization.

### Quantitation of Cas9 enrichment signal by qPCR

In the CRIST assay, quantitative real-time PCR was used to compare the enrichment signals between treatment groups. As previously described (Li et al. 2014; Zhang et al. 2014), quantitative PCR was performed using SYBR Green PCR Master (Applied Biosystems) in triplicate using a sequence detector (ABI Prism 7900HT; Applied Biosystems). The enrichment signals were calculated using threshold cycle (Ct) values standardized over the input, applying the 2**^−^**^(Δ**Ct**)^ method (Pian et al. 2018).

### RT-PCR quantitation

Mouse fibroblasts and iPSCs were collected at different stages of reprogramming, and total RNA was extracted by TRIzol reagent (Sigma-Aldrich) and stored at −80°C. cDNA was synthesized using RNA reverse transcriptase, and PCR was carried out using KlenTaq I Mix with a Bio-Rad Thermal Cycler. PCR amplification was performed for 1 cycle at 95°C for 5 min, 33 cycles at 95°C for 20 sec, 62°C for 15 sec, and 72°C for 15 sec, and 1 cycle at 72°C for 10 min. PCR products were quantified, and *Actb* was used as a PCR control. Primers used for PCR lncRNA quantitation are listed in Supplemental Table S3. For quantitative real-time PCR, the threshold cycle (Ct) values of target genes were assessed by quantitative PCR in triplicate using a sequence detector (ABI Prism 7900HT; Applied Biosystems) and were normalized over the Ct of the *Actb* control (Wang et al. 2014, 2016).

### Knockdown of the *Sox2*-interacting lncRNA

The *Sox2*-interacting lncRNAs were knocked down using lentiviral shRNAs. Briefly, four shRNAs for each lncRNA were cloned into two separate pGreenPuro vectors (#SI505A-1, SBI). Each vector contained two shRNAs that are driven by *U6* and *H1* promoters, respectively. For the control group, two random shRNAs were used in the assay. The shRNA DNA sequences are listed in Supplemental Table S3. After lentiviral transfection, iPSCs were selected by puromycin and the fate of cells was tracked by CopGFP fluorescence. After confirming the efficacy for each lentiviral vector, the cells treated with two shRNA lentiviruses were collected for the study.

### Cytosolic and nuclear fractionation assay

A cytosolic and nuclear fractionation assay was used to examine the subcellular location of lncRNAs (Chen et al. 2018). Briefly, iPSC cells were trypsinized and treated with hypotonic buffer (10 mM HEPES, pH 7.9, 1.5 mM MgCl_2_, 10 mM KCl, 0.4% Nonidet P-40). After centrifugation at 3000 rpm for 7 min, the supernatants were collected as cytosolic fractions. The pellet was resuspended and treated with hypotonic buffer twice. The final pellet was collected as the nuclear fraction. Total RNAs were purified from both fractions and were used for qPCR quantitation of lncRNAs. *U6* was used as the nuclear control and *Actb* was used as the cytosolic control.

### RNA and RNA-DNA FISH

RNA-DNA FISH was performed using a modification of the previously published method (Nath and Johnson 2009; Barakat and Gribnau 2014; Lai et al. 2016). The RNA FISH probe was prepared as a short antisense single-strand DNA that crosses the introns to probe for the mature RNAs (Zhao et al. 2019). Briefly, the short single-strand DNA probe was synthesized using a ratio of 1:50 primers that cover the intron splicing site with Dig labeling dNTP MIX (Roche #11277065910). The *Sox2* DNA probe was prepared from *Sox2* BAC clone RP23-213M12 (Bacpacresources.org) with biotin-14-dCTP using a nick translation kit (Sigma-Aldrich) according to the manufacturer's protocol. After sequential RNA and DNA FISH, slides were counterstained with DAPI. The FISH images were collected with Chroma filter sets using an Olympus BX41 upright microscope (100×, oil, 1.4 NA) equipped with a motorized z-axis controller (Prior) and Slidebook 5.0 software (Intelligent Imaging Innovations). The optical sections were collected using a NoNeighbor algorithm operating within Slidebook 5.0. The geometric centers of foci were quantitated in Slidebook 5.0. Images were merged to confirm the colocalization of the DNA-RNAs.

### Activation of core stem cell factor gene promoters by luciferase assays

The luciferase reporter assay was performed to examine the effect of the *Sox2* promoter-interacting lncRNAs on the activity of stemness genes. Three luciferase reporter vectors were constructed by cloning the promoters of *Pou5f1*, *Sox2*, and *Nanog* into the pGL3-basic reporter vector. For the *Pou5f1* reporter vector, a 4-kb DNA fragment covering the promoter and exon 1 was amplified from the mouse genomic DNA by PCR primers: JH4684 5′-TATCGATAGGTACCGTCTGTGAGGAGGTGGCTGAACTC-3′ and JH4687 5′-ATCGCAGATCTCGAGCTCCTCGGGAGTTGGTTCCAC-3′. The PCR product was then ligated into the KpnI/XhoI site at the multiple cloning site of the vector. A 2.5-kb DNA fragment was amplified from the mouse *Sox2* promoter with primers: JH6219 5′-TATCGATA**GGTACC**CCAGAGATTCGTGTTGAGCGTA-3′ and JH6223 5′-ACCGGAATGCC**AAGCTT**CTCCGTCTCCATCATGTTATACATGT-3′. The PCR products were cloned into the KpnI/HindIII site to construct the pSox2-2.5K vector. An Addgene *Nanog* reporter vector (#16337) containing a 2.5-kb *Nanog* promoter was used for this study.

Two *Sox2* promoter-interacting lncRNAs *Platr10* and *Snhg14* were cloned into the pCMV-RsRed-Puro vector. A luciferase assay was performed by cotransfecting 293T cells with the expression vector plasmid DNAs, the luciferase reporter vectors, and the *Renilla* luciferase plasmid control using Lipofectamine 3000 (Invitrogen). Forty-eight hours after transfection, firefly and *Renilla* luciferase activities were measured consecutively with the dual-luciferase reporter system (Promega) using a luminometer (modulus single tube multimode reader, Turner Biosystems). All luciferase assays were repeated three times with three culture replicates.

### The *Sox2*-interacting lncRNAs promote reprogramming

Lentiviruses carrying two *Sox2* promoter-interacting lncRNAs *Platr10* and *Snhg14* were packaged in 293T cells. Control lentiviruses carried the pCMV-RsRed-Puro empty vector (Vector) and the pCMV-800bpCT-RsRed-Puro control vector. Briefly, OG2 MEFs were first transfected with the lncRNA and control lentiviruses. After selection by puromycin, 15,000 lentivirus-transfected MEFs were seeded in 12-well plates and were reprogrammed in KSR iPSC medium containing 2 µg/mL doxycycline (DOX) (Zhuang et al. 2018). The medium was changed every other day. The iPSC colonies were immunostained with rabbit anti-NANOG Antibody (A300-397A, Bethyl, 1:500 dilution). Positive iPSC colonies per field were recorded between groups (Chen et al. 2012).

### Immunohistochemical staining of stem cell markers

As previously described (Chen et al. 2012), immunohistochemical staining was used to examine stem cell markers for iPSC colonies. Briefly, iPSCs were fixed by 4% paraformaldehyde, permeabilized with 0.1% Triton X-100/PBS containing 3% BSA, and incubated with primary antibodies overnight at 4°C. After washing with PBS, secondary antibodies were added for immunostaining, including rabbit anti-NANOG (sc33759, Santa Cruz Biotechnology, 1:100 dilution) and rabbit anti-OCT4 (AB3209, Millipore, 1:100 dilution). Samples were subsequently incubated with Cy3 or Alexa Fluor 488 labeled secondary antibodies (ab6939 and ab150077, Abcam) and were counterstained with Hoechst 33258 (Invitrogen). Fluorescence images were acquired with a Zeiss AxioCam Camera. In addition, pluripotency was also examined by a Fluorescent Mouse ES/iPS Cell Characterization kit (SCR077, Millipore) following the protocol provided by the manufacturer.

### Embryoid body differentiation

The embryoid body (EB) assay was used to examine the dynamic expression of core stem cell factor genes (Chen et al. 2012, 2016). Briefly, iPSCs were trypsinized by Collagenase Type IV (Invitrogen). Cell clumps were collected and were transferred to a new 60-mm dish in ES medium without LIF. After being maintained in floating culture for 3 d, cells were seeded in 0.1% gelatin-coated six-well plates in DMEM/F12 containing 20% FBS. After spontaneous differentiation, cells were collected at different time points for gene expression analysis using quantitative PCR.

### Statistical analysis

The data are expressed as mean ± SD. Data were analyzed using SPSS software (version 16.0; SPSS, Inc.). A Student's *t*-test or one-way ANOVA (Bonferroni test) was used to compare statistical differences for variables among treatment groups. Results were considered statistically significant at *P* < 0.05.

## Data access

All raw and processed sequencing data generated in this study have been submitted to the NCBI Gene Expression Omnibus (GEO; https://www.ncbi.nlm.nih.gov/geo/) under accession numbers GSE107945 and GSE116605.

## Supplementary Material

Supplemental Material
