# Supplementary material for: Profiling the long noncoding RNA interaction network in the regulatory elements of target genes by chromatin in situ reverse transcription sequencing
Source: Genome Res. 2019 Sep;29(9):1521–32. doi: 10.1101/gr.244996.118 (PMC6724666; doi:10.1101/gr.244996.118)
Supplement: Supplemental Material [file supp_29_9_1521__index.html]

Profiling the long noncoding RNA interaction network in the regulatory elements of target genes by chromatin in situ reverse transcription sequencing — Supplemental Material 

# Profiling the long noncoding RNA interaction network in the regulatory elements of target genes by chromatin in situ reverse transcription sequencing

## Supplemental Material

- Supplemental\_Materials.pdf
