# Supplementary material for: Profiling the long noncoding RNA interaction network in the regulatory elements of target genes by chromatin in situ reverse transcription sequencing
Source: Genome Res. 2019 Sep;29(9):1521–32. doi: 10.1101/gr.244996.118 (PMC6724666; doi:10.1101/gr.244996.118)
Supplement: Supplemental Material [file supp_gr.244996.118_Supplemental_Materials.pdf]

Profiling the long noncoding RNA interactome in the regulatory elements of target genes by chromatin *in situ* reverse transcription sequencing

Shilin Zhang[1*], Yichen Wang[1*], Lin Jia[1*], Xue Wen[1*], Zhonghua Du[1*], Cong Wang[1,2], Yajing Hao[3], Dehai Yu[1], Lei Zhou[1], Naifei Chen[1], Jingchen Chen[1,2], Huiling Chen[4], Hui Zhang[7], Ilkay Celik[2], Günhan Gülsoy[6], Jianjun Luo[3], Baoming Qin[7], Xueling Cui[8], Zhonghui Liu[8], Songling Zhang[1#], Miguel A. Esteban[7#], Ferhat Ay[5#], Wei Xu[1#], Runsheng Chen[3#], Wei Li[1#], Andrew R. Hoffman[2#], Ji-Fan Hu,[1,2#], Jiuwei Cui[1#]

[1] Key Laboratory of Organ Regeneration and Transplantation of Ministry of Education, The First Hospital of Jilin University, Changchun, Jilin 130061, P.R. China

[2] Stanford University Medical School, VA Palo Alto Health Care System, Palo Alto, CA 94304, USA

[3] CAS Key Laboratory of RNA Biology, Institute of Biophysics, Chinese Academy of Sciences, Beijing 100101, China, P.R. China

[4] Department of Endocrinology, Xiangya Hospital, Central South University, Changsha, Hunan, P.R. China

[5] La Jolla Institute for Allergy and Immunology, La Jolla, California 92037, USA

[6] Google Inc., Mountain View, CA 94043, USA

[7] Guangzhou Institutes of Biomedicine and Health, Chinese Academy of Sciences, Guangzhou, Guangdong 510530, P.R. China

[8] Department of Immunology, College of Basic Medical Sciences, Jilin University, Changchun, Jilin 130021, P.R. China

SUPPLEMENTAL TABLES

Table S1. Top fifty RNAs that interact with the *Sox2* promoter

gene_id	gene_name	Description	locus	ips-Cas9-Sox2	p-value	Qvalue
ENSMUSG00000075014	Gm10800	predicted_gene_10800	2:98666546-98667301	289804	0	0
ENSMUSG00000097312	Gm26870	predicted_gene_26870	9:3000281-3038316	270000	6.44E-42	7.91E-41
ENSMUSG00000075015	Gm10801	predicted_gene_10801	2:98662236-98664083	143433	0	0
ENSMUSG00000095891	Gm10717	predicted_gene_10717	9:3000281-3038316	92795.7	0	0
ENSMUSG00000095280	Gm21738	predicted_gene_21738	14:19415856-19418930	57206.9	0	0
ENSMUSG00000095547	Gm10719	predicted_gene_10719	9:3000281-3038316	46582.1	0	0
ENSMUSG00000096519	Gm10721	predicted_gene_10721	9:3000281-3038316	43657.5	0	0
ENSMUSG00000095186	Gm10718	predicted_gene_10718	9:3000281-3038316	31472.1	0	0
ENSMUSG00000074564	Gm10720	predicted_gene_10720	9:3000281-3038316	31106.8	0	0
ENSMUSG00000091028	Gm10722	predicted_gene_10722	9:3000281-3038316	27463.2	0	0
ENSMUSG00000096201	Gm10715	predicted_gene_10715	9:3000281-3038316	16700.7	0	0
ENSMUSG00000096385	Gm11168	predicted_gene_11168	9:3000281-3038316	15173.2	7.88E-48	1.04E-46
ENSMUSG00000079600	Gm17604	predicted_gene_17604	X:165003764-165004829	5908.06	0	0
ENSMUSG00000096736	Gm17535	predicted_gene_17535	9:3000281-3038316	5623	1.84E-139	5.39E-138
ENSMUSG00000106234	Gm43535	predicted_gene_43535	9:110951544-110984066	4916.22	0	0
ENSMUSG00000106128	Gm43520	predicted_gene_43520	3:116818814-116818978	4282.81	0	0
ENSMUSG00000066810	Gm10181	predicted_gene_10181	9:25089421-25089490	3189.07	7.88E-285	4.23E-283
ENSMUSG00000086807	Platr21	pluripotency_associated_transcript_21	X:139240225-139357738	3030.74	0	0
ENSMUSG00000106512	Gm43078	predicted_gene_43078	3:35413832-35414000	2993.53	0	0
ENSMUSG00000064945	Rny3	RNA_Y3_small_cytoplasmic_associated_with_Ro_protein_	6:47781623-47781725	2928.53	0	0
ENSMUSG00000100826	Snhg14	small_nucleolar_RNA_host_gene_14	7:59384596-59411173	2302.75	0	0
ENSMUSG00000106628	Gm43558	predicted_gene_43558	3:134357291-134357501	2101.37	0	0
ENSMUSG00000031654	Cbln1	cerebellin_1_precursor_protein	8:87468853-87472592	1612.6	0	0
ENSMUSG00000026790	Odf2	outer_dense_fiber_of_sperm_tails_2	2:29889220-29931746	1557.75	0	0
ENSMUSG00000090592	Gm17571	predicted_gene_17571	9:13444033-13446753	1514.2	1.22E-227	5.47E-226
ENSMUSG00000107569	RP24-472K15.2	-	6:139140769-139140933	1442.72	0	0
ENSMUSG00000103322	Gm37404	predicted_gene_37404	3:62123206-62123565	1385.88	0	0
ENSMUSG00000085328	Gm17131	predicted_gene_17131	5:127781629-128433077	1321.62	4.40E-225	1.95E-223
ENSMUSG00000094472	Gm21897	predicted_gene_21897	16:15317457-15318957	1296.27	0	0
ENSMUSG00000055491	Pprc1	peroxisome_proliferative_activated_receptor_gamma_coactivator_related_1	19:46032592-46072915	1259.52	0	0
ENSMUSG00000107564	RP23-447L9.3	-	6:4364231-4364395	1154.18	1.46E-320	9.60e-319
ENSMUSG00000061331	Gm17132	predicted_gene_17132	5:127781629-128433077	1152.04	0	0
ENSMUSG00000102889	Gm37282	predicted_gene_37282	9:98748598-98820087	1130.13	5.97E-314	3.74e-312
ENSMUSG00000104961	Gm43835	predicted_gene_43835	5:64563770-64563919	1110.41	2.63E-308	1.58E-306
ENSMUSG00000103837	Gm37614	predicted_gene_37614	9:88826603-88826813	1091.98	0	0
ENSMUSG00000105108	Gm42687	predicted_gene_42687	3:115881580-115934361	1074.39	2.63E-298	1.50E-296
ENSMUSG00000094856	Gm21962	predicted_gene_21962	3:137671523-137672540	1041.57	3.40E-125	9.05E-124
ENSMUSG00000103234	Gm37158	predicted_gene_37158	Y:16691329-16697033	1018.71	3.45E-87	7.06E-86
ENSMUSG00000031626	Sorbs2	sorbin_and_SH3_domain_containing_2	8:45507787-45827906	1001.77	9.26E-278	4.90E-276
ENSMUSG00000079024	Gm21961	predicted_gene_21961	15:64979543-65014904	972.827	8.02E-270	4.12E-268
ENSMUSG00000103547	Gm37665	predicted_gene_37665	9:90863158-90863307	908.515	7.70E-252	3.76E-250
ENSMUSG00000097695	Gm26905	predicted_gene_26905	8:57774051-58914298	897.85	7.53E-198	2.98E-196
ENSMUSG00000078984	Gm11027	predicted_gene_11027	12:116275385-116275612	855.115	0.046782165	0.136957613
ENSMUSG00000103070	Gm37903	predicted_gene_37903	1:137528849-137531230	830.087	4.47E-29	4.61E-28
ENSMUSG00000105285	Gm43238	predicted_gene_43238	5:62428718-62429077	815.998	0	0
ENSMUSG00000054256	Msi1	musashi_RNA_binding_protein_1	5:115429598-115455698	776.785	4.06E-215	1.71E-213
ENSMUSG00000031098	Syt8	synaptotagmin_VIII	7:142434849-142440396	762.104	4.54E-211	1.87E-209
ENSMUSG00000085659	Gm12325	predicted_gene_12325	11:71702309-71704344	722.557	5.26E-200	2.09E-198
ENSMUSG00000104174	Gm37701	predicted_gene_37701	2:12106631-12312315	674.842	1.19E-218	5.07E-217

Table S2. Top fifty pathways of the *Sox2* promoter-interacting RNAs

pathway_ID	PATHWAY_DES	target_gene_in_this_pathway	target_gene_in_all_pathway	all_gene_in_this_pathway	all_gene_in_all_pathway	rich_factor	Pvalue	Qvalue
mmu01100	Metabolic pathways	143	1121	1286	7510	0.7449539	1	1
mmu05200	Pathways in cancer	56	1121	397	7510	0.9450001	1	1
mmu04151	PI3K-Akt signaling pathway	53	1121	345	7510	1.0291794	0.7722307	1
mmu04080	Neuroactive ligand-receptor interaction	47	1121	285	7510	1.1048093	0.451655	1
mmu04024	cAMP signaling pathway	45	1121	198	7510	1.5225854	0.0069541	0.6583235
mmu04144	Endocytosis	45	1121	282	7510	1.0690493	0.5963987	1
mmu04015	Rap1 signaling pathway	40	1121	215	7510	1.2463955	0.1532033	1
mmu04010	MAPK signaling pathway	40	1121	253	7510	1.0591898	0.6489145	1
mmu04020	Calcium signaling pathway	38	1121	180	7510	1.4143126	0.0353502	1
mmu05205	Proteoglycans in cancer	38	1121	203	7510	1.2540703	0.1516755	1
mmu04014	Ras signaling pathway	37	1121	229	7510	1.0824319	0.5657807	1
mmu04360	Axon guidance	36	1121	175	7510	1.3781573	0.0552274	1
mmu04810	Regulation of actin cytoskeleton	36	1121	217	7510	1.1114171	0.4703018	1
mmu04072	Phospholipase D signaling pathway	35	1121	143	7510	1.6397073	0.0052294	0.7425711
mmu04022	cGMP-PKG signaling pathway	35	1121	172	7510	1.363245	0.0662948	1
mmu04510	Focal adhesion	35	1121	203	7510	1.1550648	0.3512603	1
mmu04740	Olfactory transduction	30	1121	1082	7510	0.1857498	1	1
mmu04724	Glutamatergic synapse	28	1121	115	7510	1.6311523	0.0121716	0.6913462
mmu04261	Adrenergic signaling in cardiomyocytes	28	1121	149	7510	1.2589431	0.1975834	1
mmu04390	Hippo signaling pathway	28	1121	154	7510	1.2180683	0.2600805	1
mmu04530	Tight junction	27	1121	140	7510	1.2920224	0.1631335	1
mmu04060	Cytokine-cytokine receptor interaction	27	1121	265	7510	0.6825779	1	1
mmu05166	HTLV-I infection	27	1121	283	7510	0.639163	1	1
mmu05016	Huntington,s disease	26	1121	196	7510	0.8886927	1	1
mmu04725	Cholinergic synapse	24	1121	113	7510	1.4228762	0.0783957	1
mmu04919	Thyroid hormone signaling pathway	24	1121	117	7510	1.3742309	0.1080171	1
mmu04921	Oxytocin signaling pathway	24	1121	158	7510	1.0176267	0.8286659	1
mmu05202	Transcriptional misregulation in cancer	24	1121	176	7510	0.9135512	1	1
mmu04550	Signaling pathways regulating pluripotency of stem cells	23	1121	140	7510	1.1006117	0.5581837	1
mmu04062	Chemokine signaling pathway	23	1121	197	7510	0.7821606	1	1
mmu04068	FoxO signaling pathway	22	1121	134	7510	1.0998975	0.5644964	1
mmu04630	Jak-STAT signaling pathway	22	1121	161	7510	0.9154426	1	1
mmu05152	Tuberculosis	22	1121	176	7510	0.8374219	1	1
mmu00230	Purine metabolism	22	1121	178	7510	0.8280127	1	1
mmu04911	Insulin secretion	21	1121	86	7510	1.635894	0.0259343	1
mmu04713	Circadian entrainment	21	1121	98	7510	1.4355805	0.0884218	1
mmu04750	Inflammatory mediator regulation of TRP channels	21	1121	126	7510	1.1165626	0.5245433	1
mmu04910	Insulin signaling pathway	21	1121	142	7510	0.9907527	1	1
mmu04514	Cell adhesion molecules (CAMs)	21	1121	164	7510	0.8578469	1	1
mmu05164	Influenza A	21	1121	171	7510	0.8227303	1	1
mmu05169	Epstein-Barr virus infection	21	1121	217	7510	0.6483267	1	1
mmu04520	Adherens junction	20	1121	74	7510	1.810642	0.010298	0.7311571
mmu05032	Morphine addiction	20	1121	93	7510	1.4407259	0.0919673	1
mmu04070	Phosphatidylinositol signaling system	20	1121	96	7510	1.3957032	0.1192444	1
mmu04972	Pancreatic secretion	20	1121	101	7510	1.326609	0.1763637	1
mmu04723	Retrograde endocannabinoid signaling	20	1121	102	7510	1.3136031	0.1896189	1
mmu04066	HIF-1 signaling pathway	20	1121	110	7510	1.2180683	0.3180193	1
mmu04931	Insulin resistance	20	1121	111	7510	1.2070947	0.336799	1
mmu05145	Toxoplasmosis	20	1121	112	7510	1.1963171	0.3561545	1
mmu04728	Dopaminergic synapse	20	1121	132	7510	1.0150569	0.832685	1

Table S3. Oligonucleotide primers used for PCR

ID	Oligo Name	Oligo sequence	Product size
RT-PCR			
Platr10	SJ229	CACTGCTGGTTTGGAGCTCCAT	121bp
	SJ230	TGGGACAGTCTCTGGATGGCCT	
Pou5f1	J648	CAGAGGATGGCTGAGTGGGCTGTA	142bp
	J649	TCAACCCTCAAGGTCCTCTCAC	
Sox2	J652	GAAGTTTGGAGCCCGAGGCTTAAG	164bp
	J653	TGCACGGCCCTGCGCGGAGATCTG	
Nanog	J650	GCTTAGGGGGCATCCTCTGATCTA	133bp
	J651	ACCTGAAACTTCCCACTAGAGAT	
Peln4	JH4902	TGGCAGCCTCTGAGTTGGGCA	122bp
	JH4093	CTTGTCCTTGACTTGGTAACATC	
Actb	J880	CAGGTCATCACCATTGGCAATGAGC	135bp
	J881	CGGATGTCCACGTCACACTTCATGA	
U2	JH1055	ATCTGTTCTTATCAGTTTAATATCTG	151bp
	JH1056	GGGTGCACCGTTCCTGGAGGTAC	
shRNAs			
Platr10			
1		TTCTGTGTATCTGTTGAGCCAGGCA	
2		CCTGCTGCCTGTCAATCCAAATGTA	
3		CTGCCAGCATCTGACTAAGATAGAT	
4		TAGTATGGCTGTCCTCGGAGAGGCT	
Snhg14			
1		TTCAAACAATTTGTGAAGAATGTTA	
2		TCTCAGTATTGTCATAAGTATGAAG	
Control (shCT)		GCAGCAACTGGACACGTGATCTTAA	
CRIST Cas9 gRNA			
Sox2			
pSox2-1		GGGGTTGAGGACACGTGCTG	
pSox2-2		GAGCCAATATTCCGTAGCAT	
5’-gCT		GAGAGGTACAATGGTCACTC	
Pou5f1			
p*Pou5f1*-1		GAACATTCAATGGATGTTTT	

p*Pou5f1*-2	GTGTGAGGGGATTGGGGCTC
5’-gCT	GAAGTGGGATGATCCTCTGA
FLI1	
p*FLI1*-1	GATGAGTGGGTGAGCCGCTC
pFLII-2	GTGGACCCCGTCATTGTTCC
5’-gCT	GTATGACTGGTCTCCTTATA
IGF2	
p*IGF2*-1	GCCTTGCGTTCCCCAAAATT
p*IGF2*-2	GTCGCCGGCTTCCAGGTAAG
5’-gCT	GTTCTACGGTGTTATGTCAA
Control (gCT)	GAAGTGGGATGATCCTCTGA

LncRNA PCR			
Spilr32	JH4117	GAGTAGTGCATTAACTAATGG	112bp
	JH4118	CCACCTCTAGTTTTCAGAACTG	
Spilr33	JH4119	GAGATGGTGGCTAAACCAGG	102bp
	JH4120	GAGGCACTGGAGACCATGATG	
Spilr22	JH4096	CTCAGCCTAACGTCTCCAAGC	126bp
	JH4097	CTGATGCTCCAGCTTCTCAGAC	
Spilr5	JH4016	CCGATTGCTGCTGCTTCTACTT	118bp
	JH4017	CCAGGCTCAGGTTAGCTCCACT	
Spilr7	JH4020	TGGCCTTCAGACTCTATCATCCA	113bp
	JH4021	GCCTCATGTTGACGTTCATCCA	
Spilr8	JH4082	CTCGGAGAGGCTCTGCCAGCAT	119bp
	JH4083	GAGCTGTCAGAACATGCATGTC	
Spilr9	JH4027	CACTGCTGGTTTGGAGCTCCATG	122bp
	JH4028	GTGGGACAGTCTCTGGATGGCCT	
Snhg14	JH5188	CTGCATCAGCAAAGAGTAGTGC	159bp
	JH5189	TGGCTGTAGAACCAACGGCTA	
Snurf	JH5190	TCATTGGCACCTTCAAGGCT	208bp
	JH5191	GGCACACGAGCAATGCCAGT	
Actb	J880	CAGGTCATCACCATTGGCAATGAGC	135bp
	J881	CGGATGTCCACGTCACACTTCATGA	
Sox2			
5-Ct	JH4405	CATAAGTAGTTCCCCACTGA	135bp
	JH4406	AATGCAAGGGCTTTGCACGC	
pSox2	JH4373	GAGCCAATATTCCGTAGCATG	136bp
	JH4374	CGCTGGGGAACCTTTGTATC	
Off	JH5877	AGCCATCCTGTCCTCCGCCTG	128bp
target	JH5878	CTGCACGGAAGGTCACGATG	

A. CRIST-seq

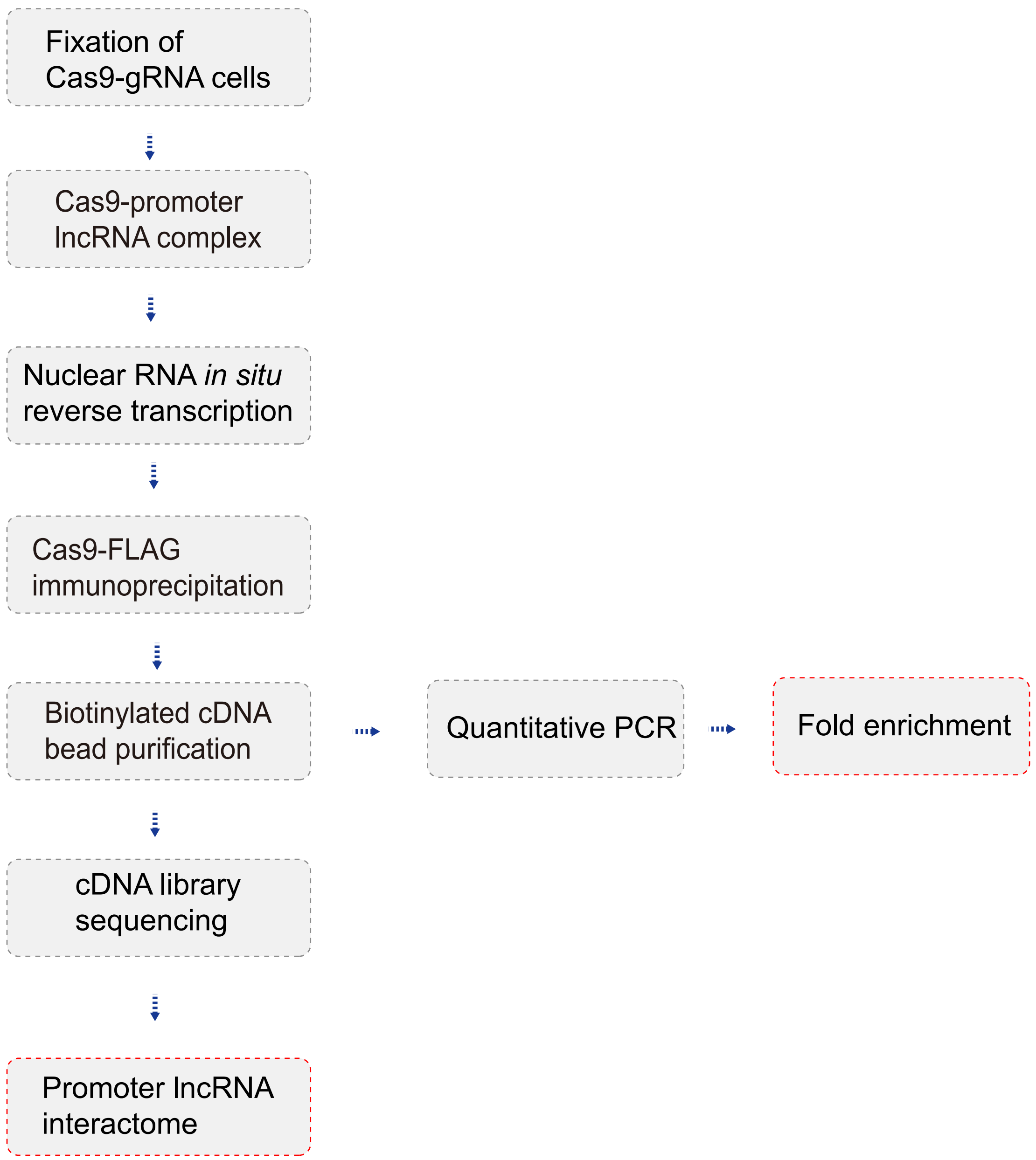

Figure S1. CRIST-seq assay to map the promoter lncRNA interactome.
Chromatin-RNA in situ reverse transcription trap sequencing (CRIST-Seq) assay. Cells were transfected with CRISPR dCas9 gRNA to target the gene promoter. The Cas9-gRNA expressing cells were crosslinked by formaldehyde to fix the promoter RNA chromatin structure. After cell membrane lysis, the nuclei were isolated and the promoter-interacting RNAs were in situ reverse transcribed into cDNAs with biotin-dCTP. The promoter biotin-cDNA chromatin complex was first immunoprecipitated by a Cas9-FLAG antibody, and the promoter-interacting biotin-cDNAs were separated from genomic DNAs by streptavidin beads. The CRIST-captured chromatin cDNAs were aliquoted for Illumina library sequencing or quantitative PCR to measure the enrichment of the identified lncRNAs to the target gene promoter.

A. *Sox2* CRIST targeting vector

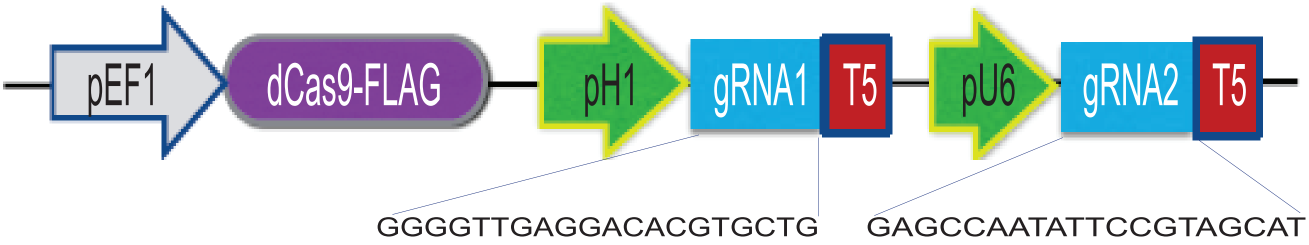

B. CRIST targeting gRNAs

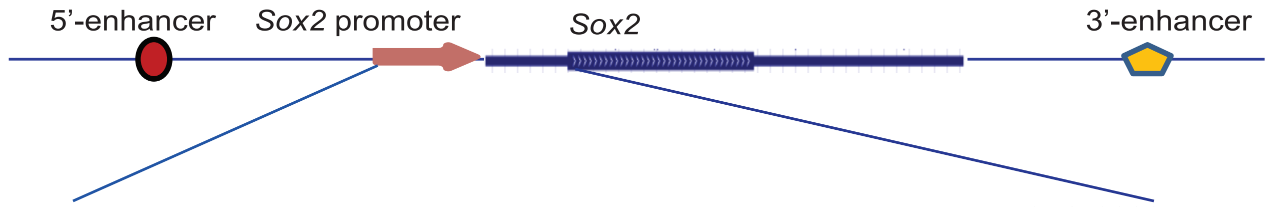


```
aatcctcttg tgtcagggtt gggagttaga aagagcggga gcagagttcc  34649200
agctttgcct ttgcaccctt tggatggggc aggttggtgg agtagggaat  34649250
taGGGGTTGA GGACACGTGC TGCGGttcct tgagccacac ctgagtcggc  34649300 gRNA1-PAM
ctaggaaaag gctgggaaca aggcctggtc ctagctctgc tctacagtca  34649350
gctacatggg cagaggactc gtgtttggga accgagccga tgacacggaa  34649400
gccaggaggg cgctgaggcc cgaggcttgg gtctaacttc tcgtctgtac  34649450
ggtgagaaga gggggtgagt gggtgctggc gacaaggttg gaagaggggc  34649500
tgcgacagag cgcagtgccg cggatgagcg cagaaacaat ggcacaccac  34649550
ctccggctct gccagcttcc tgaaatacta gttggacagt cgccctgaac  34649600
cacccatggg ccttgcccca ccctggcccc agcttcccgc gccccatcca  34649650
cccttatgta tccaagagaG AGCCAATATT CCGTAGCATG GGggaaagga  34649700 gRNA2-PAM
gctgtcgtct tggtgctgtt tacccacttc cttcgaacag gcgtgcgccg  34649750
tgacctgttg ctgaaaacgg ggggcggggg gggggatac aaaggttccc  34649800
cagcggccgg ctgcgggccc gcctccccg cgcggttcgg ggcacagcgc  34649850
tctgctgggc tcggctcggc ggcgcggcag gccccgcccc ctttcatgca  34649900
aaaccctctg gcgaggctgg gctcgggcgc aggagccggc gctcgctgat  34649950
tggccgccgg aaacccattt attccctgac agcccccatc acatggatgg  34650000
ttgtCTATta acttgttcaa aaaagtatca ggagttgtca aggcagagaa  34650050 TS +1
gagagtgttt gcaaaaaggg aaaagtactt tgctgcctct ttaagactag  34650100
ggctgggaga aagaagagga gagagaaaga aaggagagaa gtttggagcc  34650150
cgaggcttaa gcctttccaa aaactaatca caacaatcgc ggcggcccga  34650200
ggaggagagc gcctgttttt tcatcccaat tgcacttcgc ccgtctcgag  34650250
ctccgcttcc ccccaactat tctccgccag atctccgcgc agggccgtgc  34650300
acgccgaggc ccccgcccgc ggcccctgca tcccggcccc cgagcgcggc  34650350
ccccacagtc ccggccgggc cgagggttgg cggccgccgg cgggccgcgc  34650400
ccgcccagcg cccgcATGTA TAACATGATG GAGACGGAGC TGAAGCCGCC  34650450
```

Sox2 coding region

Figure S2. Location of *Sox2* Cas9-gRNAs in the CRIST assay.

A. The CRISPR Cas9 Sox2-gRNA vector used in CRIST assay. Cas9: CRISPR Cas9; gRNA1, 2: two Cas9 guiding RNAs that target the Sox2 promoter (sequences under the diagram); pEF1: the human EEF1A1 promoter; pU6: U6 promoter; pH1: human H1 promoter; T5: the TTTTT termination signal of RNA polymerase III.
B. Location of the two Cas9 gRNAs in the Sox2 promoter. The Sox2 exon 1 mRNA is shown in blue and the coding region with ATG in green. Two Cas9 gRNAs are highlighted in yellow and PAM sequences in red in the Sox2 promoter region. TS+1: transcription initiation site.
C.

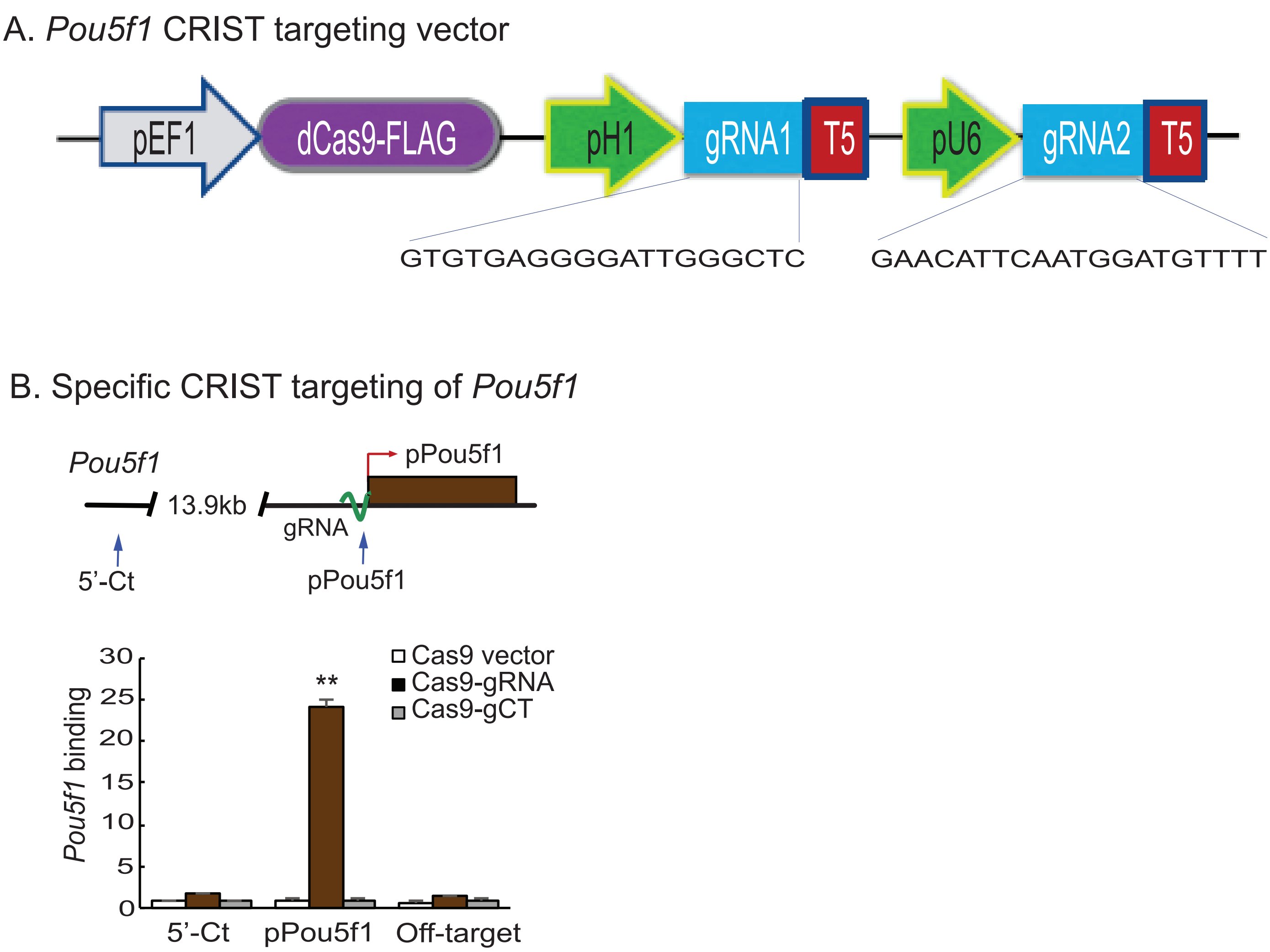

Figure S3. Specific targeting of the mouse *Pou5f1* promoter in the CRIST assay.

A. The CRISPR Cas9 Pou5f1-gRNA vector. gRNA1, 2: two Cas9 guiding RNAs that target the Pou5f1 promoter (sequences under the diagram).
B. Specific CRIST targeting of the mouse Pou5f1 promoter. pPou5f1: the targeting site in the Pou5f1 promoter where the Cas9 gRNAs are designed; 5'-Ct: the Pou5f1 control site that is 13.9 kb away from the pPou5f1 target site. Cas9 Vector: cells that were treated with the Cas9 control vector that lacks the gRNAs; Cas9-gRNA: cells that were targeted by both Cas9 and Pou5f1 gRNAs; Cas9-gCT: cells that were treated with the random control gRNA vector. Off-target: a CRIST control site that is 33.8 kb upstream of the housekeeping gene GAPDH. The chromatin complex was immunoprecipitated with a Cas9-FLAG antibody and an IgG control antibody. CRIST signals were quantitated by real-time PCR using specific primers derived from the pOct4 targeting site, 5'-Ct control site and off-target site. All data shown are mean ±SEM from three independent experiments by normalization over the IgG control. ** $p<0.01$ as compared with Vector and gCT controls.

A. *IGF2* CRIST targeting vector

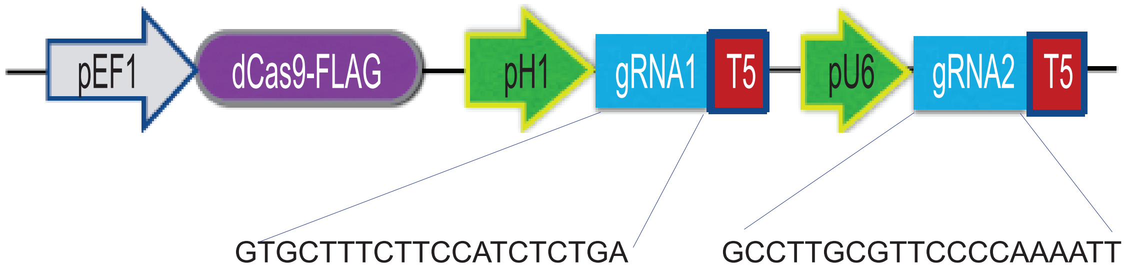

B. Specific CRIST targeting of IGF2

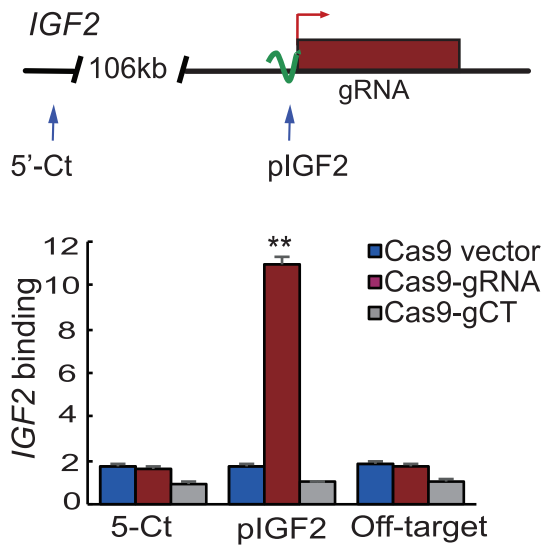

Figure S4. Specific CRIST targeting of the human *IGF2* imprinting promoters.

A. The CRISPR Cas9 IGF2-gRNA vector. gRNA1, 2: two Cas9 guiding RNAs that target the IGF2 imprinting promoters (P2-P4, sequences under the diagram).
B. Specific CRIST targeting of the growth factor IGF2 promoter. The human IGF2 has four promoters and nine exons. The promoter P1 is not imprinted and it drives the biallelic expression of the growth factor from exons 1, 2, 3, 7, 8, and 9. In contrast, its promoters P2-P4 are imprinted and are expressed exclusively from the paternal allele. In human tumors, however, this imprinting mechanism is dysregulated and causes biallelic expression of the mitogen that promotes tumor growth. We used the CRIST assay to examine the imprinted P2 promoter. pIGF2: the site in the IGF2 promoter where the Cas9 gRNAs are designed; 5'-Ct: IGF2 control site that is 106 kb away from the pIGF2 target site. Vector: cells that were treated with the Cas9 control vector that lacks the gRNAs; Cas9-gRNA: cells that were targeted by both Cas9 and IGF2 gRNAs; Cas9-gCT: cells that were treated with the random control gRNA vector. Off-target: a CRIST control site that is 33.8 kb upstream of the housekeeping gene GAPDH. The chromatin complex was immunoprecipitated with a Cas9-FLAG antibody and an IgG control antibody. CRIST signals were quantitated by real-time PCR using specific primers derived from the pIGF2 targeting site, 5'-Ct control site and off-target site. All data shown are mean±SEM from three independent experiments by normalization over the IgG control. ** $p<0.01$ as compared with Vector and gCT controls.

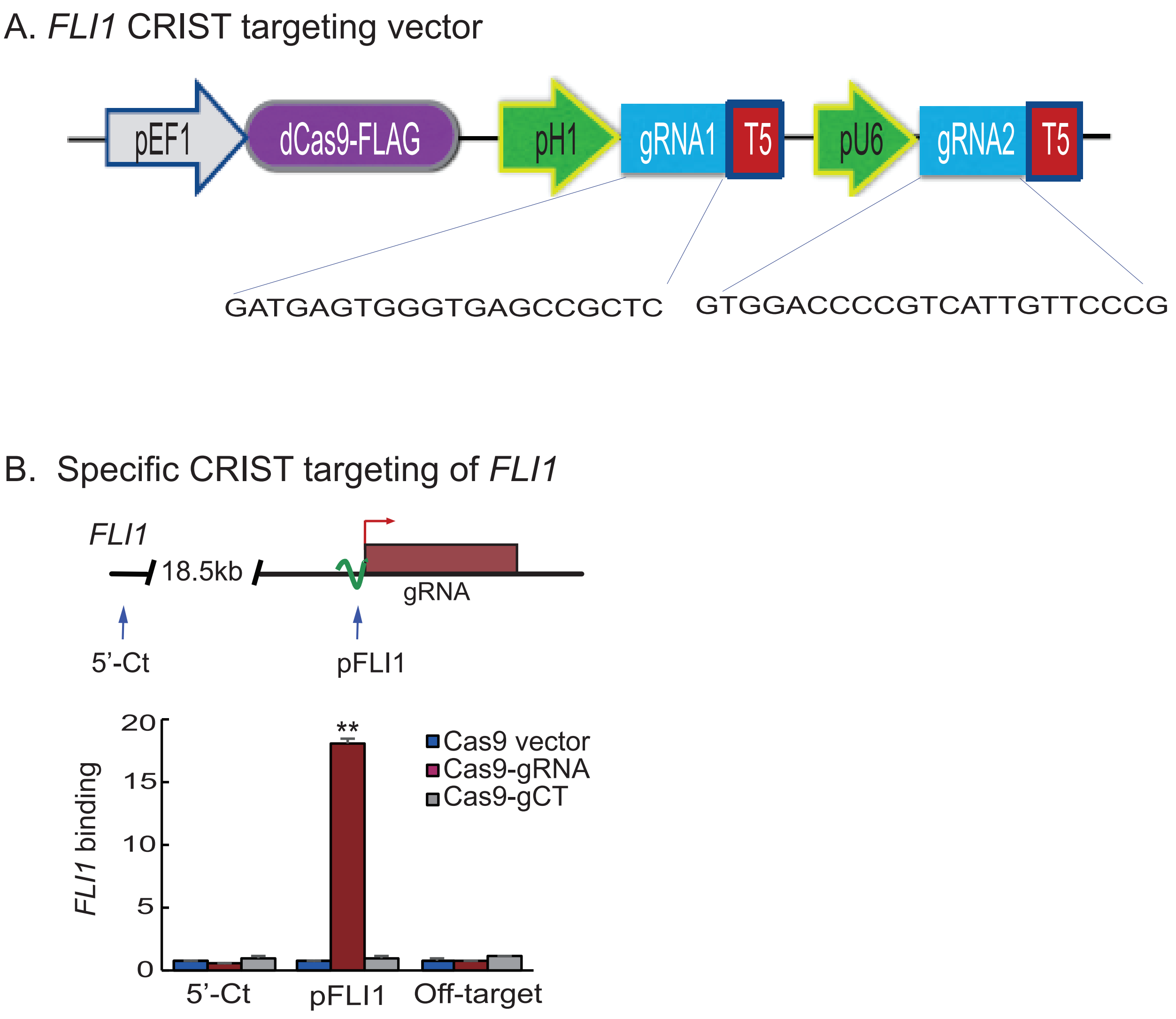

Figure S5. Specific CRIST targeting of the human *FLI1* promoter.

A. The CRISPR Cas9 FLI1-gRNA vector. gRNA1, 2: two Cas9 guiding RNAs that target the FLI1 promoter (sequences under the diagram).

B. Specific CRIST targeting of the oncogenic FLI1 promoter. pFLI1: the site in the FLI1 promoter where the Cas9 gRNAs are designed; 5'-Ct: FLI1 control site that is 18.5 kb away from the pFLI1 targeting site. Vector: cells that were treated with the Cas9 control vector that lacks the gRNAs; Cas9-gRNA: cells that were targeted by both Cas9 and FLI1 gRNAs; Cas9-gCT: cells that were treated with the random control gRNA vector. Off-target: a CRIST control site that is 33.8 kb upstream of the housekeeping gene GAPDH. The chromatin complex was immunoprecipitated with a Cas9-FLAG antibody and an IgG control antibody. CRIST signals were quantitated by real-time PCR using specific primers derived from the pFLI1 targeting site, 5'-Ct control site and off-target site. All data shown are mean±SEM from three independent experiments by normalization over the IgG control. ** $p<0.01$ as compared with Vector and gCT controls.

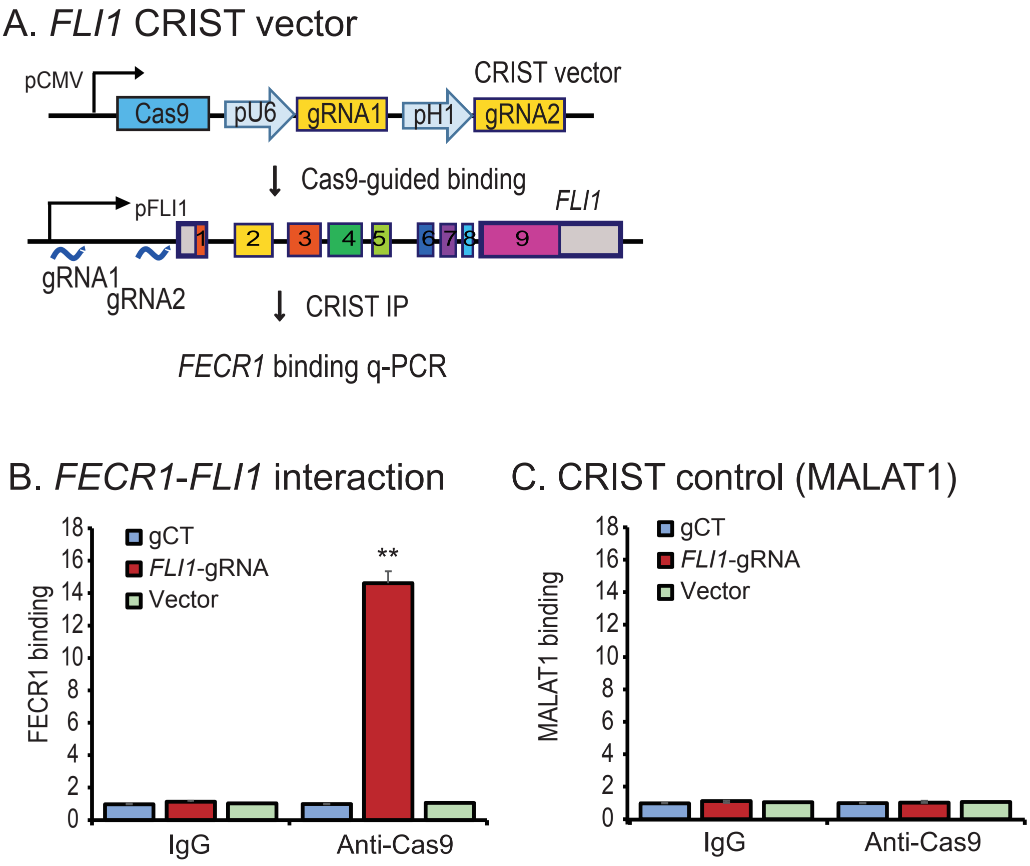

Figure S6. The *FECR1* circRNA-*FLI1* promoter interaction as a positive CRIST control.

A. The FECR1-FLI1 CRIST vector. FECR1 is a known circRNA that binds to the FLI1 promoter and regulates its activity in cis. We thus used it as a CRIST positive control. Two Cas9 FLI1 gRNAs are transcribed by U6 and H1 promoters, respectively, and guides the dCas9 to the FLI1 promoter. The FLI1 CRIST-seq library was used to quantitate the enrichment of FECR1 circRNA in the FLI1 promoter.

B. Enrichment of FECR1 in the FLI1 promoter. Vector: cells that were treated with the Cas9 control vector that lacks the gRNAs; Cas9-gRNA: cells that were targeted by both Cas9 and IGF2 gRNAs; Cas9-gCT: cells that were treated with the random control gRNA vector; Vector: cells that were treated with the empty dCas9 vector. The enrichment of FECR1 in the FLI1 promoter was quantitated by qPCR. For comparison, the value of the IgG group was set as 1. ** $p<0.01$ as compared with the IgG control group.

C. The nuclear lncRNA MALAT1 is used as the FLI1 CRIST negative control. The binding of MALAT1 to the FLI1 promoter was quantitated by qPCR and was standardized over the IgG group.

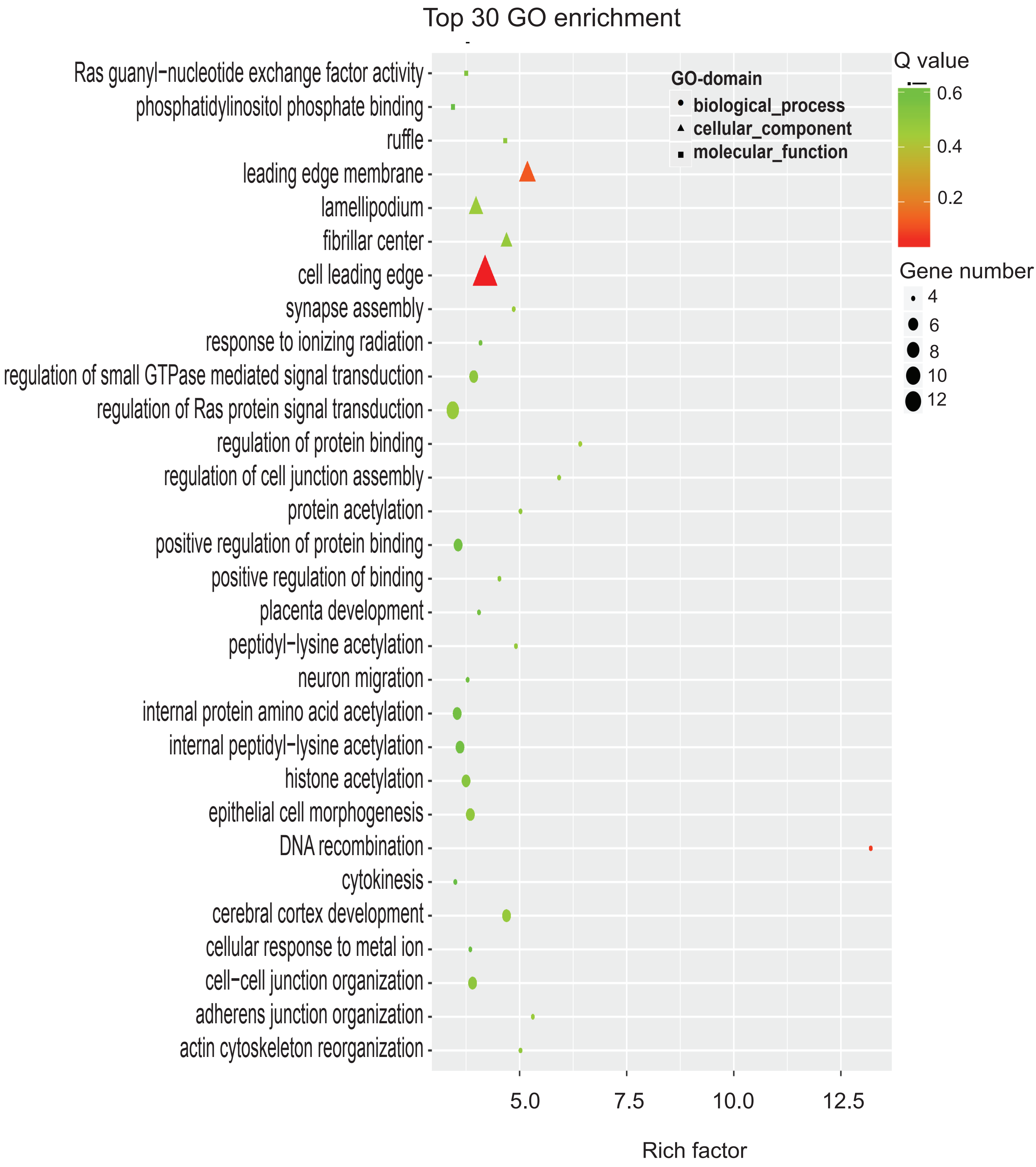

Figure S7. The top 30 GO enriched target genes of the *Sox2*-interacting RNA.
The Go enrichment was analyzed using the ggplot2 package (http://had.co.nz/ggplot2/). The top 30 enriched target genes are selected based on the enrichment score and q-value.

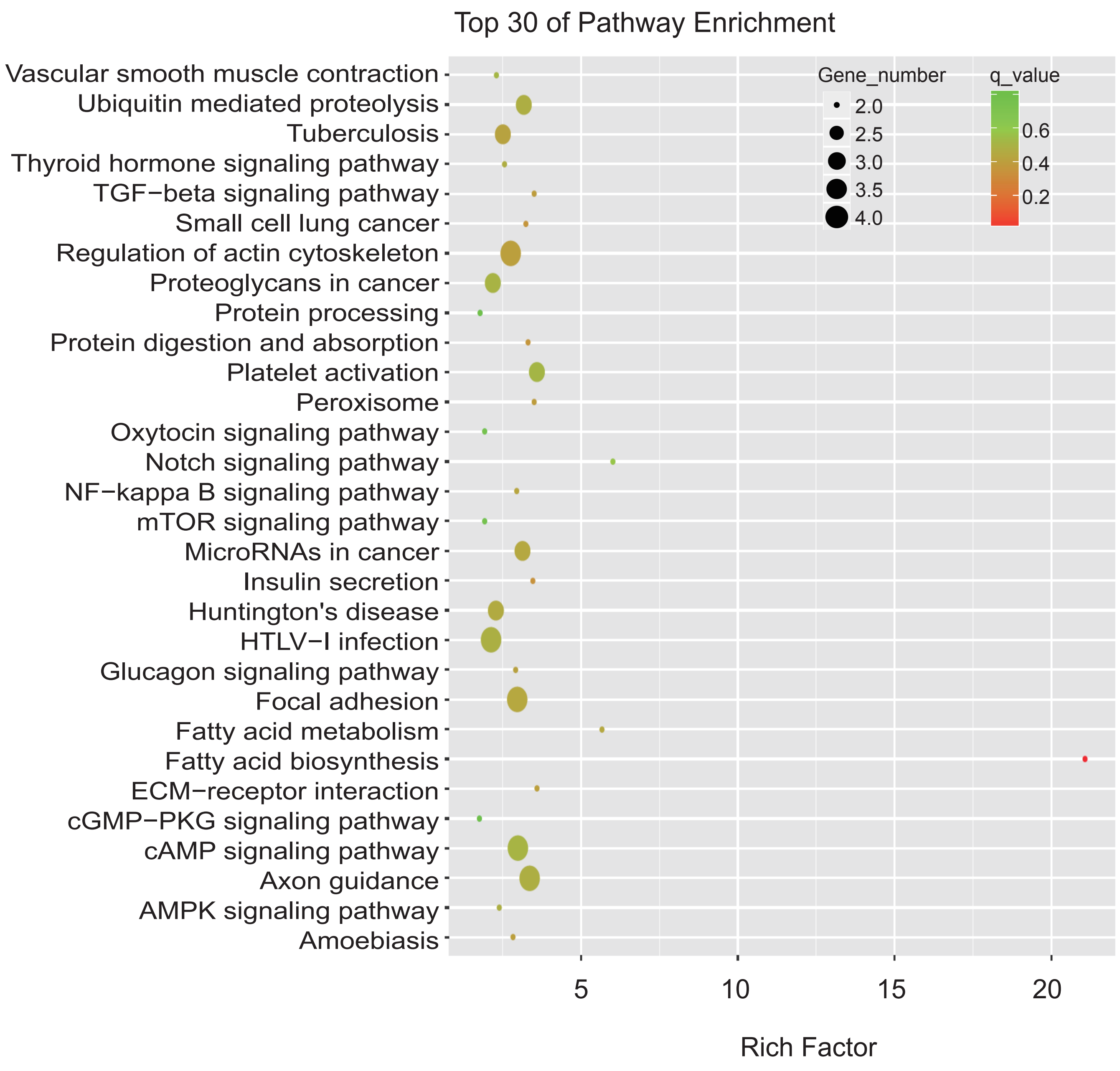

Figure S8. The top 30 KEGG pathways of the *Sox2*-interacting RNAs.
The scatterplot of the top 30 enriched KEGG pathways. KEGG: Kyoto Encyclopedia of Genes and Genomes; rich factor: the ratio of the number of DEGs and the number of all the unigenes in the pathways; Q-value: the corrected p-value.

A. Differentially expressed *Sox2* CRIST lncRNAs

Gene_id	Gene_name	Locus	Fold	p-value	q-value
ENSMUSG00000073291	Gm10491	X:7899356-7908351	14300200.0	1.22681E-38	1.43657E-36
ENSMUSG00000102064	Gm28625	9:84890544-84890749	13446000.0	1.98227E-36	2.15038E-34
ENSMUSG00000065999	Gm13154	4:147553276-147585198	1089.6	1.19663E-07	2.4399E-06
ENSMUSG00000099370	Platr10	3:75647445-75655731	987.3	3.65379E-20	2.19044E-18
ENSMUSG00000003153	Slc2a3	6:122727808-122801640	350.0	8.5813E-107	3.1165E-104
ENSMUSG00000031995	St14	9:31089401-31131853	181.8	0.003987661	0.032151481
ENSMUSG00000106628	Gm43558	3:134357291-134357501	108.9	0	0
ENSMUSG00000008489	Elavl2	4:91250762-91400785	41.9	1.5543E-06	2.66593E-05
ENSMUSG00000068686	Cd59b	2:104069848-104091187	41.8	1.53644E-06	2.65163E-05
ENSMUSG00000105270	Gm42863	5:145504096-145507054	31.1	2.65617E-11	8.45757E-10
ENSMUSG00000087306	A230004M16Rik	11:41710341-42000857	25.2	0.002198209	0.019179984
ENSMUSG00000023140	Reg2	6:78395622-78408106	22.6	5.1177E-06	8.08094E-05
ENSMUSG00000053411	Cbx7	15:79891658-79971119	19.7	1.57911E-12	5.69364E-11
ENSMUSG00000087267	4933427J07Rik	2:128955670-128957861	14.2	1.11677E-10	3.30404E-09
ENSMUSG00000061544	Zfp229	17:21730794-21769342	11.8	4.14055E-05	0.000544947
ENSMUSG00000097695	Gm26905	8:57774051-58914298	11.5	4.75322E-60	9.37881E-58
ENSMUSG00000097283	Gm26686	1:128770598-128774421	8.4	0.003341667	0.027383526
ENSMUSG00000101776	Gm28268	X:21751378-21761733	8.4	3.35742E-09	8.43022E-08
ENSMUSG00000094856	Gm21962	3:137671523-137672540	7.9	1.12718E-23	7.86794E-22
ENSMUSG00000035299	Mid1	X:169685198-170005736	7.3	1.68227E-12	6.03954E-11
ENSMUSG00000105285	Gm43238	5:62428718-62429077	7.1	3.58084E-52	5.78918E-50
ENSMUSG00000102994	Gm37767	1:161942085-162479943	6.8	9.63652E-05	0.001197231
ENSMUSG00000097171	Gm17644	1:12667562-12673090	6.3	0.000421727	0.004477993
ENSMUSG00000061331	Gm17132	5:127781629-128433077	5.8	3.4147E-06	5.54203E-05
ENSMUSG00000028693	Nasp	4:116601051-116627941	5.8	1.46116E-45	2.06865E-43
ENSMUSG00000030309	Caprin2	6:148842491-148896237	5.7	0.001187481	0.011053898
ENSMUSG00000104174	Gm37701	2:12106631-12312315	5.5	3.28739E-16	1.54847E-14
ENSMUSG00000031976	Urb2	8:119910359-124345724	5.1	6.68347E-05	0.000853189
ENSMUSG00000100826	Snhg14	7:59384596-59411173	4.6	2.34342E-05	0.000326424
ENSMUSG00000075014	Gm10800	2:98666546-98667301	4.3	3.91148E-13	1.49874E-11
ENSMUSG00000048897	Zfp710	7:80024813-80094173	4.3	0.002654322	0.022350356
ENSMUSG00000036054	Sugp2	8:70220171-70279915	4.2	0.00170335	0.015271642
ENSMUSG00000103070	Gm37903	1:137528849-137531230	4.2	0.000243982	0.00277402
ENSMUSG00000094472	Gm21897	16:15317457-15318957	3.9	1.89913E-06	3.19827E-05
ENSMUSG00000075015	Gm10801	2:98662236-98664083	3.7	0.002654322	0.022350356
ENSMUSG00000078249	Hmga1-rs1	11:120762793-120764419	3.7	1.09118E-10	3.23597E-09
ENSMUSG00000031575	Ash2l	8:25816190-25847694	3.6	1.45663E-09	3.84227E-08
ENSMUSG00000085328	Gm17131	5:127781629-128433077	3.5	0.001092886	0.010401299
ENSMUSG00000009073	Nf2	11:4765844-4849536	3.2	2.12854E-05	0.000298651
ENSMUSG00000100750	Gm29084	1:61638823-62642284	3.2	9.43789E-07	1.68811E-05
ENSMUSG00000034042	Gpbp1l1	4:116557657-116600266	3.1	0.000196949	0.002290178
ENSMUSG00000079600	Gm17604	X:165003764-165004829	3.1	0	0
ENSMUSG00000057113	Npm1	11:33152286-33163206	2.8	3.293E-106	1.1788E-103
ENSMUSG00000054717	Hmgb2	8:57511842-57515999	2.7	5.11247E-13	1.92942E-11
ENSMUSG00000071497	Nutf2-ps1	19:53588203-53589067	2.6	3.04642E-05	0.000416705
ENSMUSG00000057572	Zbtb8os	4:129335694-129378116	2.6	0.000627727	0.006371083
ENSMUSG00000008450	Nutf2	8:105860633-105880402	2.6	2.34912E-06	3.92182E-05
ENSMUSG00000031715	Smarca5	8:80698506-80739497	2.0	0.00010401	0.001284561

Figure S9. Differentially expressed *Sox2* promoter-interacting RNAs.
The *Sox2* CRIST-seq RNAs were integrated with the RNA-seq >2 fold RNAs data using a VENN program (http://bioinformatics.psb.ugent.be/webtools/Venn). A cut-off threshold of peak enrichment FPKM >50 was arbitrarily set to select CRIST-Seq RNAs. The RNAs are listed in the order of expression fold change of iPSCs over fibroblasts.

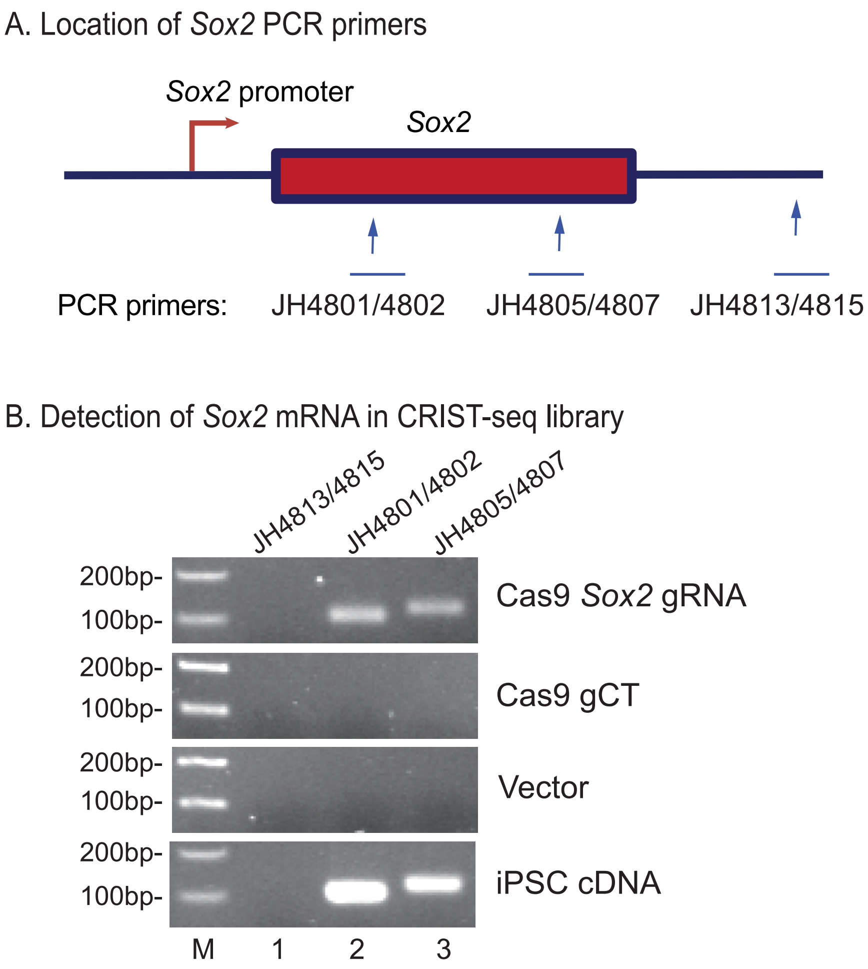

Figure S10. Detection of the *Sox2* pre-mRNA in the CRIST-seq library.
The abundance of the Sox2 pre-mRNA in the Cas9 Sox2-gRNA CRIST-seq library was detected by two primer sets from the Sox2 coding sequence. The third pair of PCR primers (JH4813/JH4815) from the downstream sequence was used as the negative control. The iPSC cDNA was used as the positive PCR control. Using this more sensitive PCR, we detected the presence of Sox2 pre-mRNA in the CRIST-seq products. As expected, no signals were detected in the Cas9 gCT and the Vector control groups.

A. *Snhg14* RNA-seq data

Gene_id	Gene_name	Locus	PSC_FPKM	FIB_FPKM	Fold	p-value	q-value
ENSMUSG00000100826	Snhg14	7:59384596-59411173	42.22	9.18	4.598299	2.34342E-05	0.000326424

B. *Snhg14* IGV

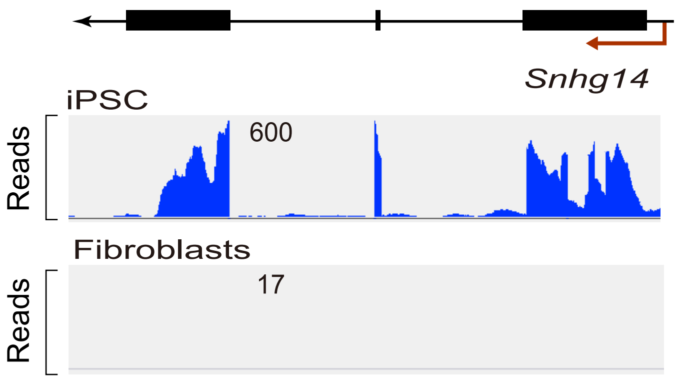

C. *Snhg14* sequence (NR_015456)

```
   1 attgtggatt tcaatttata ttctggttta caacttataa agatagtatt tcatttataa
  61 ttcagttaat tctgcaatct caatgttatg actaaattca atttgtggta aatacatatc
 121 ctgtaacttt tctaaatgtt ttaatatgtt tatatatctt taagctattt taaaattttg
 181 tatattttca tctagtatta ttaaaggtaa atacctagta tagtgtacga aaaaacagcc
 241 tgtatcacat taaaagcaat caagagaaat taaaatcaga gttaggatta atgagtaaca
 301 tttatcccag cacctttcaa gattatacac agctccgtac tcagtttgtg gacagcctga
 361 gctcatttgg ccttgtgtca aaagaaagtg tgaaaacaga aaaactgaac acatcccttc
 421 aacacaaaag gaattgtaag aatatgtatt atttgttgca attttagagt tatctcctaa
 481 tatttcatca tttgtgattc tcaaatgtaa tttgattttt tttgttggac agtaagatag
 541 ccaaatacca gatctacgaa aagaacataa ggattgcagc ttaaggtgaa tgtaatttaa
 601 cagtgaaata ttctctagaa gaggggtgtt gtattataaa ttaaaaaaag aaacaagttg
 661 ccttcttgta gacatacttt agttcatact aaaagagata gttaagaaat tcctgattat
 721 gcatatgttg tgtcattaag gaaattaata cttttgtatc ataatatgta ttattttaca
 781 gtaataattg aagatttgtg caagtcaatt aaactataca tgcttggaaa ttgccacatg
 841 ggaccatctt gaatgaacag tttatgcaaa cagaaaacat gttcaggaag aatgttaaaa
 901 ttttaacact tgtaaataga aagaagatga catttgcctg tctttgtaat ttatgagggt
 961 atttgcaaat aagcatcaaa acaagaagaa aatattaaaa agacattttg tgccaggaca
1021 tcatggtagt gctggattgc ctatccaact ctgtataaag gtttttcaaa caatttgtga
1081 agaatgttag tatcttcaga gtcttctgca tcagcaaaga gtagtgcatt aactaatgga
1141 aaagagaaga taaaatttct ggtgctcatg atgactagtc cgaacctgtg aacttctgag
1201 aaaatttaca gttctgaaaa ctagaggtgg ttaccgaatt gaatagccgt tggttctaca
1261 gccactataa tgatctgaaa agttgaatgt gttgcatgaa cactttcagg aatcataata
1321 gaactcagct aattagatag agactggaat gttgatttga agacaattac tttataaaga
1381 ttttctcatt gctgcttttg cattatccca agaagtggag acactactac atggataaga
1441 actgtatgat tgaattgact ttggaatcat ctcagtattg tcataagtat gaagtgtatt
1501 tcatggtact tttgaagtta gttatagaat tttttcaatt gatttttcta tttttcaaag
1561 ggtttaggta atatgtagca tagtctgact tgagtggtaa cattttagaa atataccagg
1621 tactgttagg gcaatattgt gtgaagattt actttatag tgcatgccaa atttgggtt
1681 ttctggtaca ttttattgga ttttatttat taatatttgt tataagagca gctgattatg
1741 ggttaagatg atagtattac agttatgtgt caaaattcta ttactttgaa ctacattggc
1801 ttggtatatc aagtctacat ttgattaaat gtagacttag caggattaat gaatttggcc
1861 attaaaacta caggaatttt agtcaacatt cctctcatat ttgtgtattt atgtatgtat
1921 gtatgtatgt acaggaattt tagtcaacat tcctctcatt tgtatatgta tgtatgtatg
1981 tatttattta tttatttatt atcaaatcat gctgttcatt tctgctcatt gtttgagttt
2041 ccatgctaat tatctattat tcataagtac atttataaat aagtctgtaa gcaggtcatc
2101 atgtatgtca gataaaatac tgtgttttct actctctgtt ttattactaa gttgtatatg
2161 aattatgcat taaatacaga atgttctctc ttaaaaaaaa aaaaaaaaaa aaaaaaaaaa
```

Figure S11. Differential expression of *Snhg14* lncRNA in reprogramming.

A. RNA-seq expression of *Snhg14* between iPSCs and fibroblasts collected in reprogramming.

B. IGV Sashimi plot of *Snhg14* lncRNA between iPSCs and fibroblasts.

C. *Snhg14* lncRNA sequence.

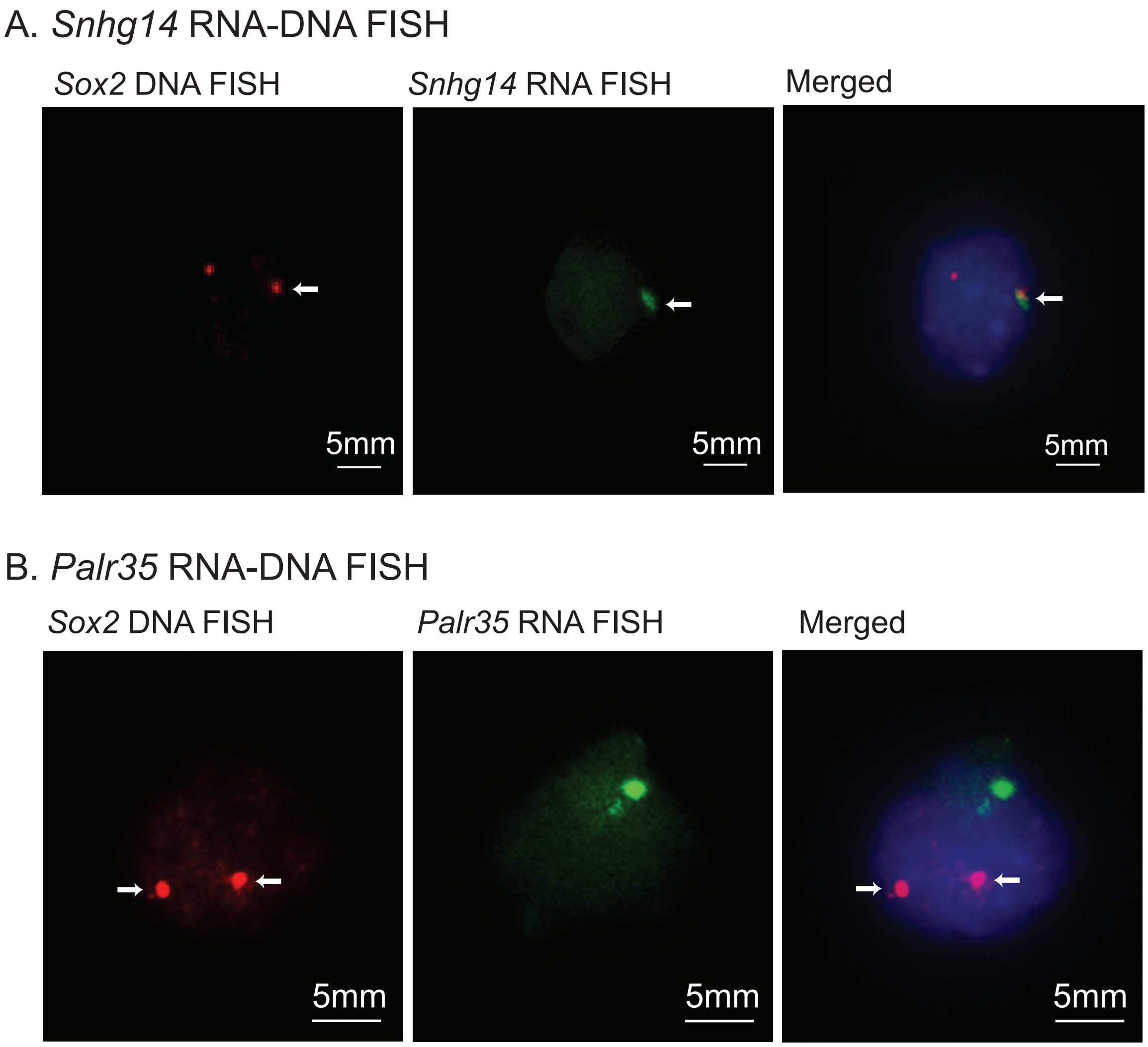

Figure S12. Interaction of lncRNAs with the *Sox2* locus by RNA-DNA FISH.

A. Snhg14 RNA-DNA FISH. The isolated iPSC clones were subjected to RNA FISH (green) using biotin-14-dCTP-labeled single strand DNA probes for Snhg14. RNA probes are designed to cover the intron region for specific staining of the mature RNAs. Slides were subsequently re-fixed and processed for DNA FISH (red) using digoxigenin-dUTP labeled BAC probe. The nuclear DNA was stained with DAPI (blue). Arrows mark the co-localization of the Snhg14 lncRNA signal with the Pou5f1 DNA signal.

B. Palr35 RNA-DNA FISH. Although lncRNA Palr35 was also differentially expressed in reprograming, it was not in the list of the Sox2 CRIST-seq. We thus used it as the RNA FISH control. As the control, RNA-FISH did not detect the co-localization of Palr35 with Sox2.

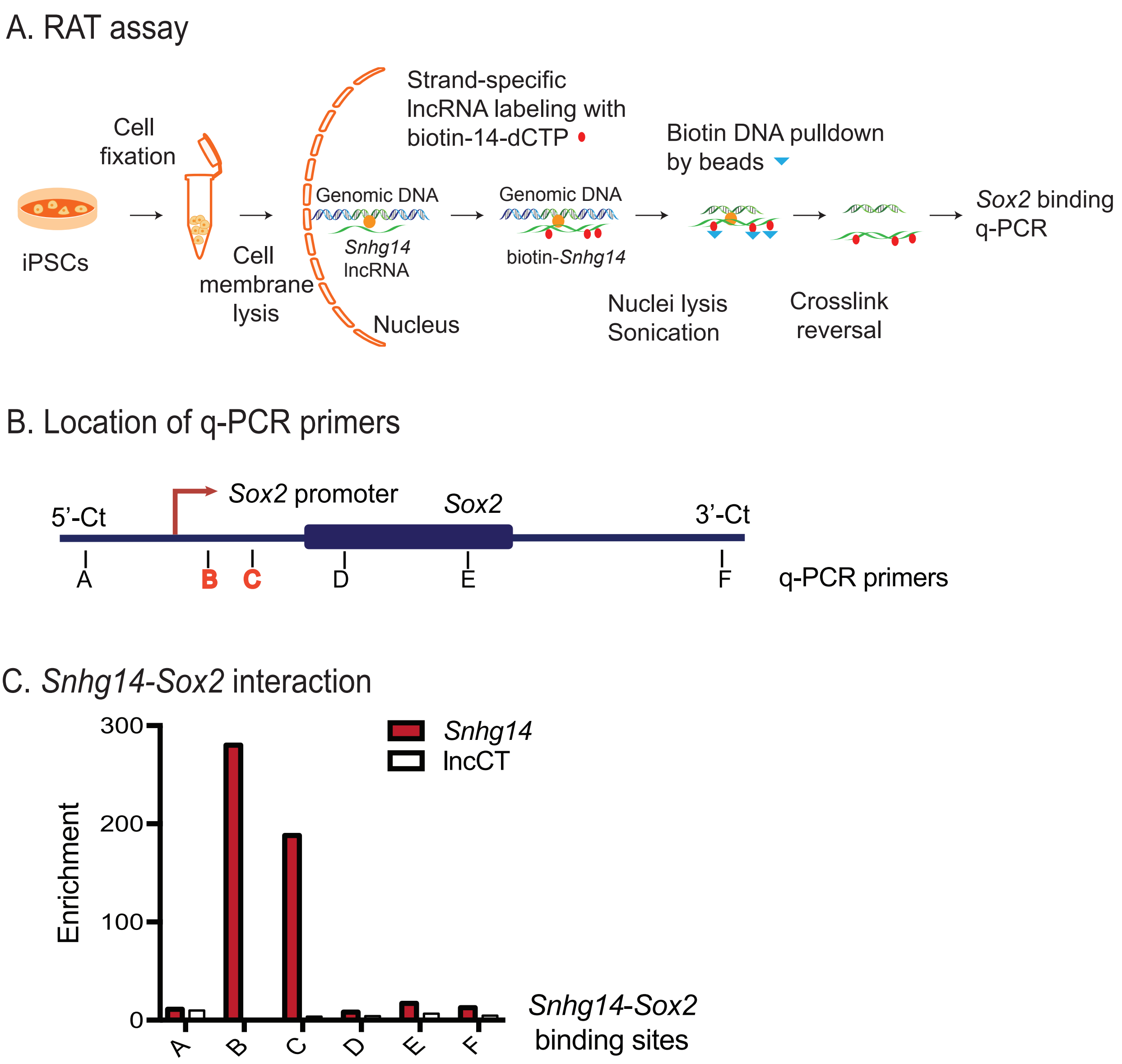

Figure S13. Validation of the *Snhg14-Sox2* interaction by the RAT assay.

A. The RAT assay. iPSCs were fixed and Snhg14 lncRNA was labelled by biotin-dCTP in the nucleus using four Snhg14-specific oligonucleotides. The random oligonucleotides were used as the RAT control. After sonication, the biotin-Snhg14-chromatin complex was pulled down by streptavidin beads. The chromatin DNA was purified for qPCR.
B. Location of qPCR primers. Primers B and C are designed from the Sox2 promoter.
C. The Snhg14-Sox2 interaction by qPCR. The Snhg14-pulled down chromatin DNAs were mapped by a series of primers in the Sox2 locus. Note the enriched signal of the Sox2 promoter (B, C sites) in the Snhg14-pulled down chromatin complex.
